# Mitochondrial Activity and Cyr1 Are Key Regulators of Ras1 Activation of *C*. *albicans* Virulence Pathways

**DOI:** 10.1371/journal.ppat.1005133

**Published:** 2015-08-28

**Authors:** Nora Grahl, Elora G. Demers, Allia K. Lindsay, Colleen E. Harty, Sven D. Willger, Amy E. Piispanen, Deborah A. Hogan

**Affiliations:** Department of Microbiology and Immunology, Geisel School of Medicine at Dartmouth, Hanover, New Hampshire, United States of America; University of Toronto, CANADA

## Abstract

*Candida albicans* is both a major fungal pathogen and a member of the commensal human microflora. The morphological switch from yeast to hyphal growth is associated with disease and many environmental factors are known to influence the yeast-to-hyphae switch. The Ras1-Cyr1-PKA pathway is a major regulator of *C*. *albicans* morphogenesis as well as biofilm formation and white-opaque switching. Previous studies have shown that hyphal growth is strongly repressed by mitochondrial inhibitors. Here, we show that mitochondrial inhibitors strongly decreased Ras1 GTP-binding and activity in *C*. *albicans* and similar effects were observed in other *Candida* species. Consistent with there being a connection between respiratory activity and GTP-Ras1 binding, mutants lacking complex I or complex IV grew as yeast in hypha-inducing conditions, had lower levels of GTP-Ras1, and Ras1 GTP-binding was unaffected by respiratory inhibitors. Mitochondria-perturbing agents decreased intracellular ATP concentrations and metabolomics analyses of cells grown with different respiratory inhibitors found consistent perturbation of pyruvate metabolism and the TCA cycle, changes in redox state, increased catabolism of lipids, and decreased sterol content which suggested increased AMP kinase activity. Biochemical and genetic experiments provide strong evidence for a model in which the activation of Ras1 is controlled by ATP levels in an AMP kinase independent manner. The Ras1 GTPase activating protein, Ira2, but not the Ras1 guanine nucleotide exchange factor, Cdc25, was required for the reduction of Ras1-GTP in response to inhibitor-mediated reduction of ATP levels. Furthermore, Cyr1, a well-characterized Ras1 effector, participated in the control of Ras1-GTP binding in response to decreased mitochondrial activity suggesting a revised model for Ras1 and Cyr1 signaling in which Cyr1 and Ras1 influence each other and, together with Ira2, seem to form a master-regulatory complex necessary to integrate different environmental and intracellular signals, including metabolic status, to decide the fate of cellular morphology.

## Author Summary

*Candida albicans* is a successful fungal commensal and pathogen of humans. It is a polymorphic organism and the ability to switch from yeast to hyphal growth is associated with the commensal-to-pathogen switch. Previous research identified the Ras1-cAMP-protein kinase A pathway as a key regulator of hyphal growth. Here, we report that mitochondrial activity plays a key role in Ras1 activation, as respiratory inhibition decreased Ras1 activity and Ras1-dependent filamentation. We found that intracellular ATP modulates Ras1 activity through a pathway involving the GTPase activating protein Ira2 and the adenylate cyclase Cyr1. Based on our data the canonical Ras1 signaling model in *C*. *albicans* needs to be restructured in such a way that Cyr1 is no longer placed downstream of Ras1 but rather in a major signaling node with Ras1 and Ira2. Our studies suggest that the energy status of the cell is the most important signal involved in the decision of *C*. *albicans* to undergo the yeast-to-hyphae switch or express genes associated with the hyphal morphology as low intracellular ATP or associated cues override several hypha-inducing signals. Future studies will show if this knowledge can be used to develop therapies that would favor benign host-*Candida* interactions by promoting low Ras1 activity.

## Introduction

*Candida albicans*, one of the most common human fungal pathogens, is an important cause of morbidity and mortality in immunocompromised individuals, particularly in patients with AIDS or those undergoing cancer chemotherapy or transplantation procedures [1]. The prolonged use of antifungal agents in such compromised populations can lead to an increase in *C*. *albicans* resistance to many currently used therapies [2]. For this reason, there is an immediate need for new treatment options that can prevent or control diseases caused by *C*. *albicans*.

In addition to being a pathogen, *C*. *albicans* is also a member of the commensal microflora of most individuals and the transition from commensal to pathogen is associated with the morphological switch from yeast to hyphal growth [3–5]. Environmental factors like 37°C, 5% CO_2_, N-acetylglucosamine, pH, and serum, induce the yeast-to-hyphae switch [6]. However, most of these signals are always present in vivo, thus we still do not understand what governs the switch from benign colonization to symptomatic infection. During host colonization *C*. *albicans* lives amidst other microbes and, both, clinical data, that suggest a link between antibiotic usage and increased risk of fungal infections, and laboratory studies indicate that *C*. *albicans* interacts with bacteria in biologically important ways [7–14]. Further studies on bacterial-fungal interaction have led to the identification of new ways by which microbes modulate *C*. *albicans* growth. For example, 3-oxo-C12-homoserine lactone, produced by the Gram-negative bacterium *Pseudomonas aeruginosa*, inhibits hyphal growth by directly inhibiting the fungal Ras1-cAMP-protein kinase A (PKA) signaling pathway, a key regulator of the yeast-to-hyphae switch in *C*. *albicans*, by blocking cAMP synthesis [15,16].

The Ras1-cAMP-PKA signaling pathway is critical for *C*. *albicans* virulence in animal models [17–19]. Ras1 is a small GTPase that exists in the cell in an inactive (GDP-bound) form and an active (GTP-bound) form whose switch is regulated by the guanine nucleotide exchange factor (GEF) Cdc25 and GTPase-activating protein (GAP) Ira2 [20]. In its GTP-bound form, Ras1 directly interacts with the adenylate cyclase Cyr1 and stimulates cAMP production [18,21,22]. The cAMP signal subsequently derepresses two PKA isoforms which promote several cellular processes [23,24]. In current models of virulence, activation of the Ras1-cAMP-PKA pathway by host-associated stimuli induces the transition from yeast-to-hyphae growth and the expression of hypha-specific virulence factors. Hyphal growth increases tissue adherence and penetration, as well as the formation of adherent biofilms on medical devices [6,25,26]. This pathway also controls genes involved in glycolysis, stress resistance, cell wall composition, and mating [6,27].

More recently, an additional class of molecules that repress hyphal growth of *C*. *albicans*, phenazines, have been identified [28,29]. While phenazines are best known as small-molecule toxins with antibiotic properties toward bacterial and eukaryotic species at high concentrations [30], recent studies have found that some phenazines (phenazine-1-carboxylic acid (PCA), phenazine methosulfate (PMS), and pyocyanin (PYO)) inhibit hyphal growth, intercellular adherence and biofilm development of *C*. *albicans* at low sub-lethal (micromolar) concentrations that are more than 100-fold below the concentrations which affect fungal survival [29]. Phenazines were found to inhibit *C*. *albicans* respiration [29], which is consistent with other published data that phenazines can impact mitochondrial activity [31–33]. Subsequent analysis indicated that the decreased ability of *C*. *albicans* to develop wrinkled colonies (consisting of hyphal and yeast cells) or robust biofilms on plastic was due to inhibition in electron transport chain activity [29]. Indeed it was shown that during the filamentation process *C*. *albicans* activated the TCA cycle, inhibited the pentose phosphate pathway, and increased mitochondrial respiration [34]. This suggests that hyphal growth in *C*. *albicans* depends on functional respiration to cover the metabolic needs of the cell which is inhibited by phenazines. Early studies with mammalian mitochondria showed that phenazines uncouple oxidative phosphorylation by shunting electrons from endogenous pathways [35–37], and this is most likely how respiration is inhibited in *C*. *albicans*. The inhibition of *C*. *albicans* filamentation by phenazines occurs despite the presence of robust fermentation pathways capable of supporting rapid growth in the absence of mitochondrial activity, and suggests communication between filamentation inducing pathways and metabolic state [29]. Indeed, in eukaryotes, it is becoming increasingly apparent that signaling pathways that sense and respond to extracellular cues often also incorporate input from the mitochondria themselves or from mitochondrially-derived molecules (like ATP and reactive oxygen species) [38,39].

In this report we show, that respiratory inhibition via genetic or biochemical manipulation decreases Ras1 activity and inhibits Ras1-dependent filamentation in *C*. *albicans*. Ras1 activation is also decreased by mitochondrial inhibition in the pathogenic *Candida* species, *Candida parapsilosis* and *Candida tropicalis*. Furthermore, utilizing a *NRG1* overexpression strain and an *efg1*/*efg1* null mutant we show that decreased Ras1 signaling in the presence of respiratory inhibitors is independent of morphological change. Filamentation was not repressed by MB in strains lacking Tup1, a hyphal growth repressor, or in a strain overexpressing Ume6, a transcription factor involved in the induction of hyphal growth indicating that the effects of MB on GTP-Ras1 can be circumvented with activation of downstream parts of the pathway. Analysis of overall metabolic changes due to respiratory inhibition shows perturbation of carbon metabolism, evidence for changes in redox state and increased AMP kinase activity (increased β-oxidation, decreased sterol levels). Subsequent analysis showed that intracellular ATP modulates Ras1 activity independent of AMP kinase. Furthermore, while the GEF Cdc25 is dispensable for decreased Ras1 signaling due to respiratory inhibition, the GAP Ira2 is necessary. In addition, the adenylate cyclase Cyr1 is essential for this signaling cascade, showing for the first time that Cyr1 affects Ras1 activation state and with that it is not just a downstream effector of Ras1. Rather Cyr1, Ira2, and Ras1 seem to form a regulatory complex that combines a multitude of signals to decide if the yeast-to-hyphae switch should take place.

## Results

### Methylene blue inhibits *C*. *albicans* filamentation by decreasing signaling through the Ras1-cAMP-PKA pathway

**Fig 1:**
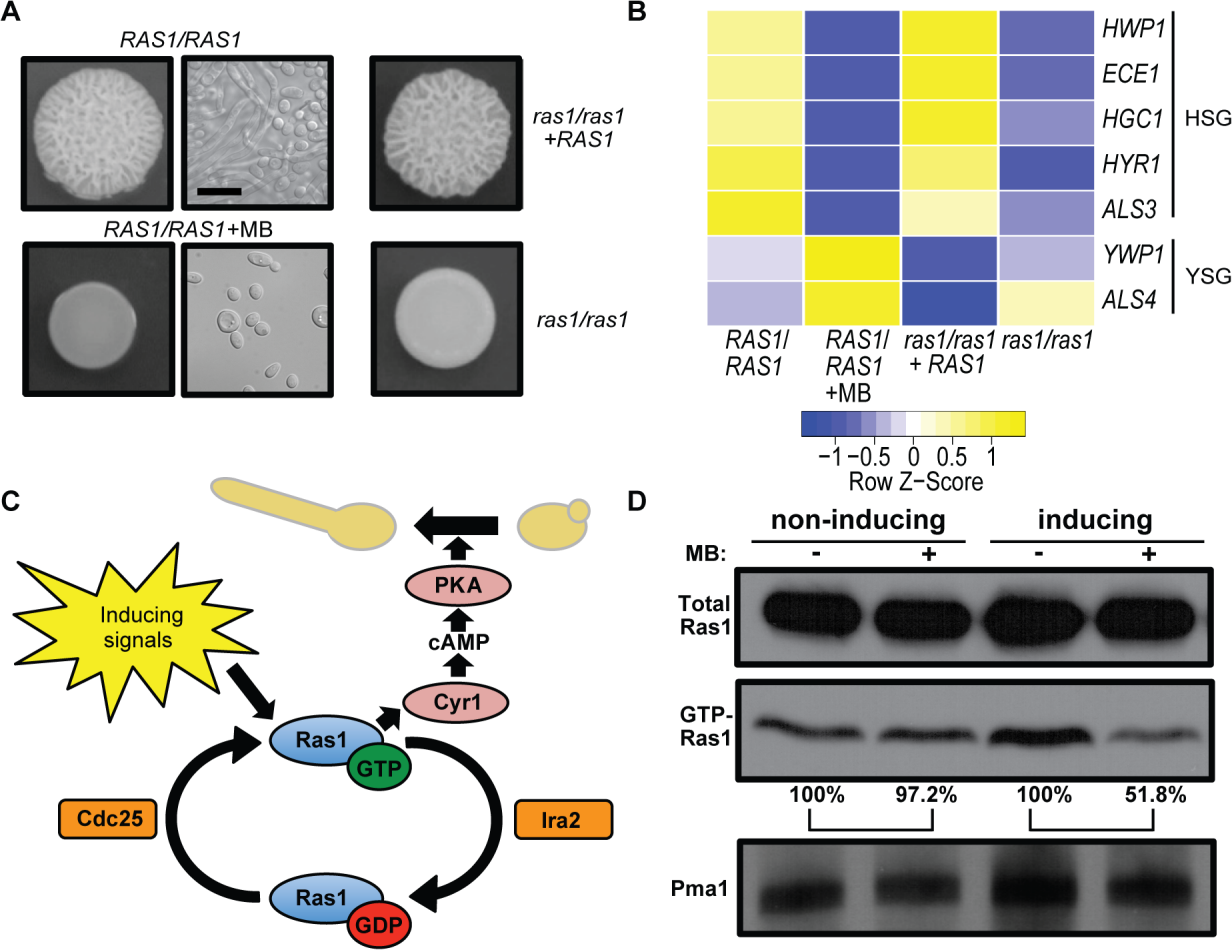
MB inhibits Ras1 activity and Ras1-dependent filamentation. **(A)** Colonies of the wild type (*RAS1*/*RAS1*, SC5314), *ras1*/*ras1*, and *ras1*/*ras1*+*RAS1* were grown with vehicle or with 1.5 μM MB on YNBAGNP at 37°C for 24 h (see S1A Fig for microscopy images of cells from all colonies). Scale bar = 10 μm. **(B)** Expression levels of hypha-specific genes (HSG) and yeast-specific genes (YSG) with and without MB or Ras1 grown under conditions listed in (A). Nanostring analysis was used to determine transcript levels. **(C)** Model of the canonical Ras1 signaling pathway in *C*. *albicans*. Ras1 cycles between an active GTP- and an inactive GDP-bound state in response to the activities of the GEF (guanine nucleotide exchange factor) and GAP (GTPase-activating protein), Cdc25 and Ira2, respectively. GTP-Ras1 binds to the adenylate cyclase, Cyr1, and triggers cAMP production. cAMP activates protein kinase A (PKA) which results in the activation of hyphae specific genes resulting in the morphological switch from yeast to hyphae. **(D)** Analysis of the total Ras1 protein and GTP-Ras1 fraction of *C*. *albicans* strain CAF2 grown on YNBAGNP at 37°C (inducing) or YNBGP at 30°C (non-inducing) for 24 h with and without MB. Pma1 levels were shown as a loading control. Percent of the GTP-Ras1/total Ras1 ratio compared to control conditions is shown.

Low micromolar concentrations of the bacterially-produced toxin, pyocyanin (PYO), or its thioanalogue, methylene blue (MB), perturb mitochondrial activity [40,41] and repress *C*. *albicans* filamentation (Fig 1A) [29]. Exposure to 1.5 μM MB, a compound used therapeutically in humans [41], caused growth solely in the yeast form as indicated by a smooth colony morphology and cellular yeast morphology as determined by microscopy. While under control conditions, *C*. *albicans* grew as a mix of yeast and hyphae in wrinkled colonies. (Fig 1A and S1A Fig panels 1 to 4). Furthermore, MB led to decreased expression of hypha specific genes and increased levels of yeast-specific transcripts (Fig 1B). Because the colony phenotype, cellular morphology, and expression profile of cells grown with MB were similar to those of the afilamentous *ras1*/*ras1* mutant (Fig 1A and 1B and S1A Fig panels 5 to 8) we sought to test the hypothesis that MB inhibits the yeast-to-hyphae switch by inhibition of Ras1 signaling, a pathway critical for *C*. *albicans* filamentation and virulence in animal models (Fig 1C) [17,18]. Examination of Ras1 protein levels in cells grown on solid medium with and without MB found that MB led to reductions in levels of active GTP-bound Ras1 (GTP-Ras1) without affecting total Ras1 levels (Fig 1D).

When MB was added to *C*. *albicans* cultures in liquid YNBAGNP medium, we observed that MB decreased clumping and increased the percentage of cells in the yeast morphology at concentrations of 3 and 6 μM, but not at 1.5 μM, a concentration that completely inhibited filamentation on solid medium (S2A Fig and Fig 1A). Analysis of the fraction of Ras1 in the GTP-bound state found that 3 and 6 μM MB also decreased the fraction of Ras1 in its active form (S2A Fig). To test if these concentrations of MB have an impact on *C*. *albicans* growth we used a strain in which the hyphal gene repressor Nrg1 is overexpressed (*NRG1-OE*). Nrg1 acts downstream of Ras1 and overexpression of this repressor prevents filamentation in the presence of hypha-inducing signals; the use of this strain makes it possible to measure growth via OD_600_ measurements in the presence of filamentation inducing signals [3,42]. The different concentrations of MB had no or only minimal impact on *C*. *albicans* growth excluding this as the reason for a decrease of GTP-Ras1 levels (S2B Fig). Because filamentation is completely inhibited by 1.5 μM MB on solid media, but filamentation is only partially suppressed even at 6 μM in liquid medium, we used colony-grown cells in subsequent assays.

We tested if MB affected GTP-binding of Ras1 in yeast growth conditions, and found that MB did not impact GTP-Ras1 levels (Fig 1D and S2C Fig). This suggests that MB selectively inhibits the increase in GTP-Ras1 that occurs in the presence of filamentation-inducing signals which include 37°C, buffering at pH 7, and the amino acids and N-acetylglucosamine in YNBAGNP medium. To determine whether the decrease of GTP-Ras1 by MB is specific to YNBAGNP, we tested another common filament-inducing condition (YPD + 5% serum at 37°C). Consistent with our findings on medium with N-acetylglucosamine, amino acids, 37°C, and neutral pH, as the hyphal growth inducers, MB led to lower GTP-Ras1 levels when grown on medium with serum and repressed filamentation (S2D Fig and S1B Fig). In liquid YPD + 5% serum, the effects of MB on morphology and GTP-Ras1 levels were modest suggesting that different media create different physiological states in *C*. *albicans* (S2E Fig). All further experiments were conducted using YNBAGNP medium as it is a defined stimulus that mimics a number of aspects of the host (pH 7, amino acids, 0.2% glucose).

**Fig 2:**
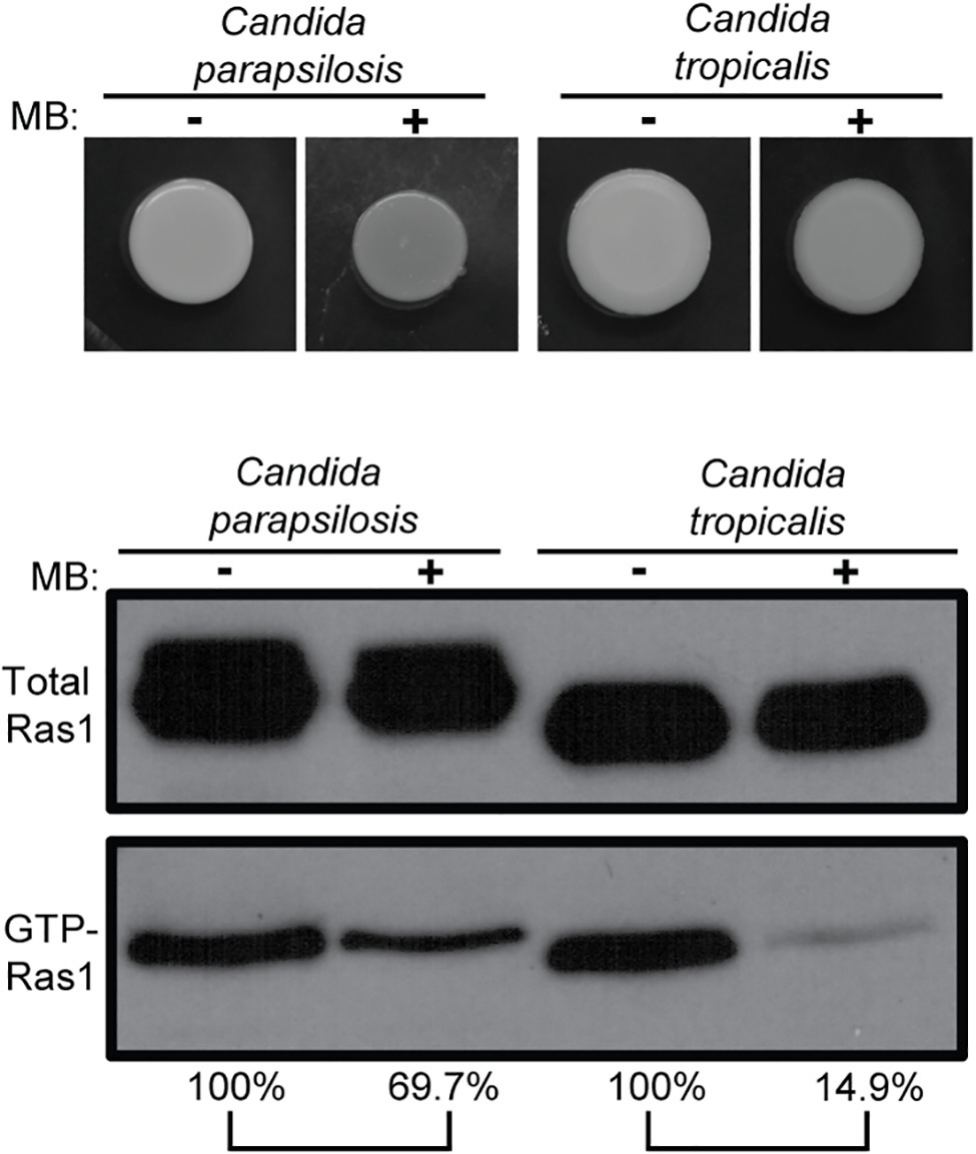
MB effects on Ras1 signaling also occur in other *Candida* species. *Candida parapsilosis* and *Candida tropicalis* were grown on YNBAGNP with and without 1.5 μM MB at 37°C for 24 h (see S1A Fig panels 9 to 12 for microscopy images of cells). Western blot analysis of total Ras1 and GTP-Ras1 levels are shown. Percent of the GTP-Ras1/total Ras1 ratio compared to control conditions is shown. Unlike *C*. *albicans*, these fungi grow as yeast in the absence and presence of MB.

To test whether a link between MB and Ras1 activation state can also be observed in other *Candida* species, we examined GTP-Ras1 levels of two other pathogenic *Candida* species, *Candida parapsilosis* and *Candida tropicalis*. These two fungal pathogens also had lower GTP-Ras1 levels on YNBAGNP with MB, indicating that Ras1 activation is also impacted by MB in other *Candida* species (Fig 2 and S1A Fig panels 9 to 12). Under these conditions, these fungi grow as yeast in the absence and presence of MB.

### Respiratory activity modulates Ras1 signaling and is independent of the morphological switch

**Fig 3:**
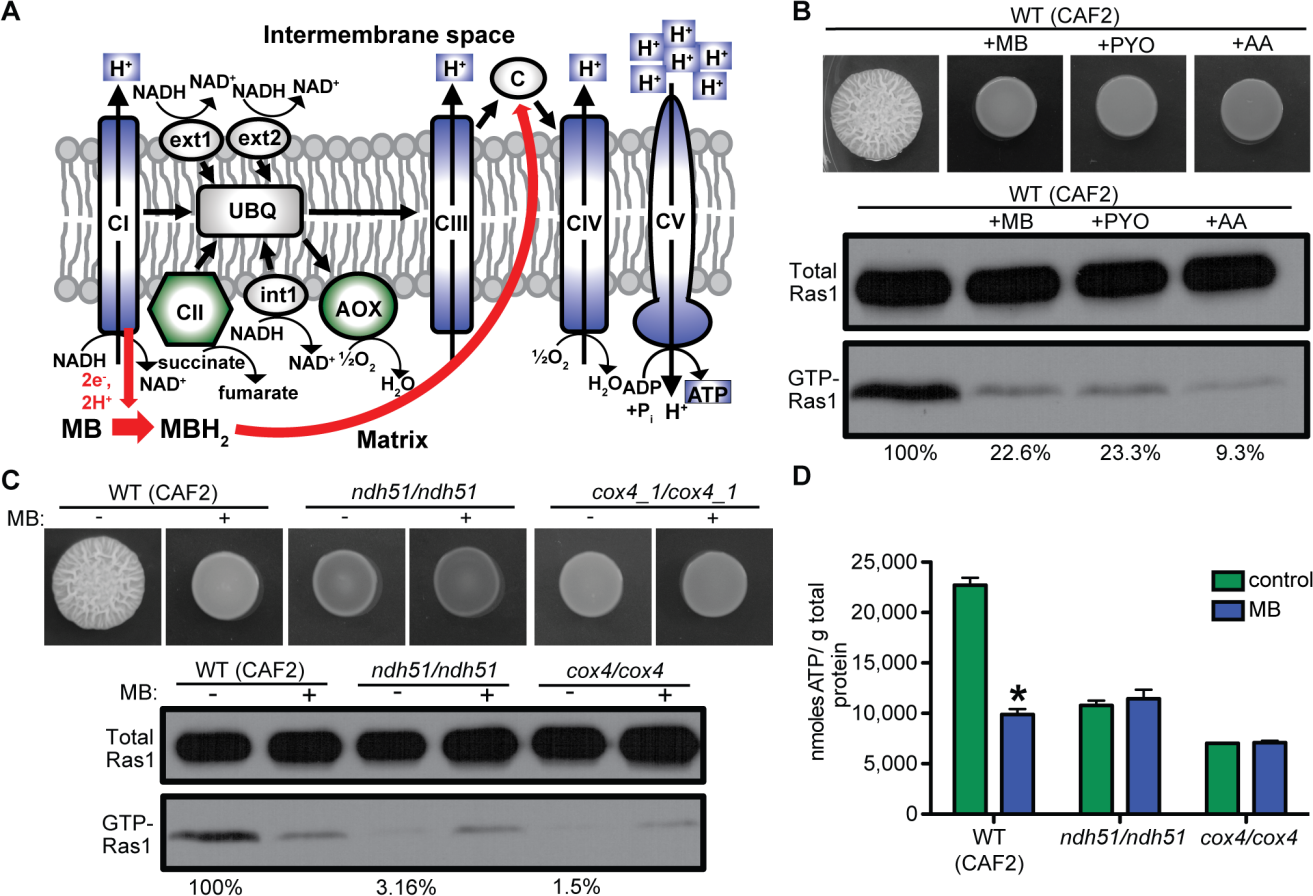
Ras1 signaling is modulated by respiratory activity and correlates with total intracellular ATP levels. **(A)** Schematic of the fungal mitochondrial electron transport chain and how it is inhibited by MB (red arrows). Normally electrons are transported to the ubiquinone pool (UBQ) from NADH by complex I (CI) or internal (int1) and external (ext1, ext2) alternative NADH:ubiquinone oxidoreductases, and from succinate by complex II (CII). From UBQ electrons are channeled through complex III (CIII), cytochrome C (c), and complex IV (CIV) or directly by the alternative oxidase (AOX) to oxygen as the terminal electron acceptor. Simultaneous to the electron transport, protons are pumped over the inner mitochondrial membrane by CI, CIII, and CIV. This proton gradient is used by the ATP synthase (CV) to generate ATP. In the presence of MB electrons are shuttled from NADH at CI directly to c reducing the proton gradient and ATP production. MBH2 reduced MB. Blue: respiratory complex important for the MB induced decrease in Ras1 signaling. Green: dispensable complex/protein for MB response. **(B)** *C*. *albicans* WT (CAF2) was grown on YNBAGNP with MB, pyocyanin (PYO), or antimycin A (AA) at 37°C for 24 h. Western blot analysis of total Ras1 and GTP-Ras1 is shown. **(C)** WT (CAF2), *ndh51*/*ndh51* (complex I), or *cox4*/*cox4* (complex IV) were grown with and without MB on YNBAGNP at 37°C for 24 h (see S1A Fig panels 13 to 16 for microscopy images of cells). Western blot analysis of total Ras1 and GTP-Ras1 is shown. Percent of the GTP-Ras1/total Ras1 ratio compared to control conditions is reported. **(D)** Intracellular ATP measurements of WT (CAF2), *ndh51*/*ndh51*, and *cox4*/*cox4* grown for 24 h on YNBAGNP at 37°C. Mean ± SD are shown. *p<0.05.

In mammalian cells, MB decreases oxidative phosphorylation potential by oxidizing NAD(P)H-dependent dehydrogenase (complex I) and directly reducing cytochrome C thereby bypassing proton transfer by complex I and complex III (Fig 3A) [43]. Inhibition of mitochondrial activity with PYO and/or the complex III inhibitor Antimycin A (AA) reduced GTP-Ras1 levels; both PYO and AA also repressed hyphal growth as previously reported (Fig 3B) [29,44]. Mutants lacking complex I (*ndh51/ndh51*) or complex IV (*cox4/cox4*) did not filament and had low levels of GTP-Ras1 under control conditions (Fig 3C and S1A Fig panels 13 to 16 for cellular morphology) [45]. GTP-Ras1 levels were not further reduced by MB, and in fact levels increased in cells exposed to MB (Fig 3C). Null mutants lacking complex II (*sdh1/sdh1*) or both alternative oxidases (*aox1-A/aox1-A aox1-B/aox1-B*), which do not participate in the formation of the proton gradient, still filamented and had high GTP-Ras1 levels (S3A and S3B Fig). MB also caused a decrease in GTP-Ras1 in the complex II and alternative oxidase mutants comparable to wild type (S3A and S3B Fig).

All three complexes important for high GTP-Ras1 levels under hypha-inducing conditions (complex I, III, and IV) pump protons into the intermembrane space for use in ATP synthesis (Fig 3A). MB reduces ATP synthesis in *C*. *albicans* as relative ATP levels were 2.3-fold lower upon growth of the wild type (WT) with MB (Fig 3D). Furthermore, while intracellular ATP in *ndh51/ndh51* and *cox4/cox4* mutants in control conditions were significantly lower than in the WT (CAF2) (Fig 3D), ATP levels were not reduced by MB (Fig 3C).

### Relationship between filamentation and GTP-Ras1 modulation by respiratory activity (MB)

**Fig 4:**
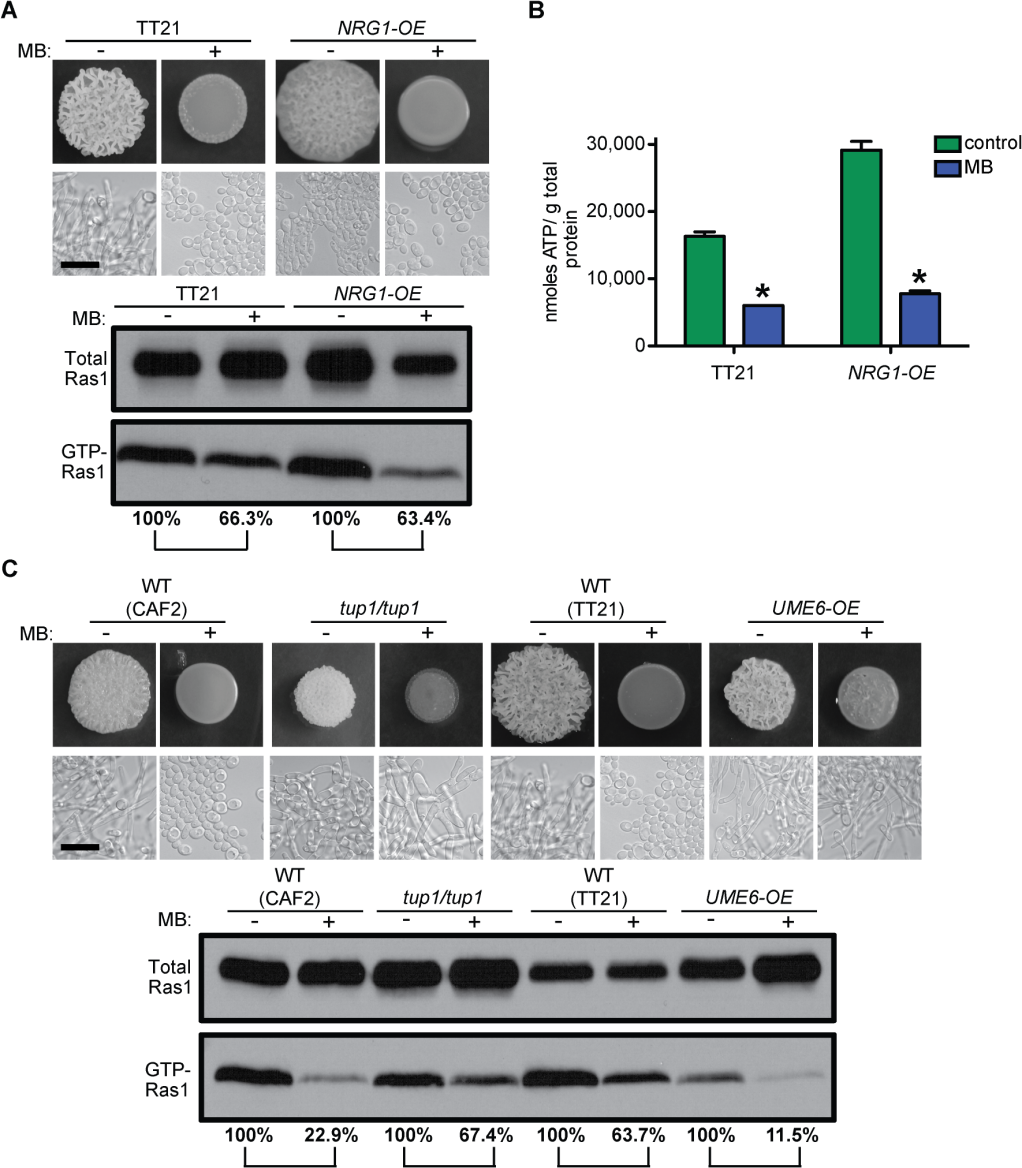
MB effects on Ras1 signaling are independent of the morphological switch and the blockage of other pathways. **(A)** WT (TT21) and the yeast locked strain *NRG1-OE* were grown with and without MB on YNBAGNP at 37°C for 24 h. Colony phenotype, cellular morphology, western blot analysis of total Ras1 protein and GTP-Ras1 levels, as well as **(B)** intracellular ATP measurements are shown. **(C)** Colony and microscopy images and western blot analysis of WT (CAF2, TT21) compared to the constitutively filamentous *tup1*/*tup1* and *UME6-OE* strains. Cells were grown for 24 h on YNBAGNP at 37°C. (B) Mean ± SD are shown. *p<0.05. (A) and (C) Percent of the GTP-Ras1/total Ras1 ratio compared to control conditions is reported. Microscopy pictures of TT21 in (A) were re-used in (C). Scale bar = 10 μm.

To determine if filamentation was required for elevated GTP-Ras1 and higher ATP, we examined the effects of MB on mutant strains that are unable to undergo the yeast-to-hyphae switch. It is well known that the transcription factor Efg1 is an essential regulator for morphogenesis in *C*. *albicans* [46]. In hyphae inducing conditions Efg1 is activated through the Ras1–cAMP–PKA pathway and induces the expression of many hyphae specific genes that are essential for the yeast-to-hyphae transition [47,48]. In addition, we tested the *NRG1* overexpression strain (*NRG1-OE*). Because both Efg1 and Nrg1 act downstream of Ras1 we hypothesized that MB effects on Ras1 should be unaffected in these strains. As expected the *efg1*/*efg1* mutant formed a smooth colony consisting of yeast cells in the presence and absence of MB (S4 Fig and S1A Fig panels 17 and 18). The *NRG1*-*OE* strain showed a weakly wrinkled colony morphology under control conditions consisting of mainly yeast cells with some elongated yeast cells and short pseudohyphae, while with MB a completely smooth colony consisting of only yeast cells was observed (Fig 4A). Subsequent western blot analysis and intracellular ATP measurements showed that both strains had less GTP-Ras1 and less ATP with MB, as the WT (Fig 4A and 4B and S4 Fig). The reduction of GTP-Ras1 in WT ranged from 26.5% to 94.5% with an average reduction of 63.6% over all experiments done; even in assays with only a 26.5% reduction in the ratio of GTP-Ras1/ total Ras1 relative to control, filamentation was repressed.

Furthermore, we tested the effects of MB on mutant strains that are constitutively filamentous due to the alteration of downstream transcriptional regulators of hyphal growth to determine if the effects of MB were upstream in the hyphal growth pathway and if hyphal growth could be reactivated in the presence of MB. Loss of the hyphal gene repressor Tup1 or overexpression of the transcription factor Ume6 have been previously shown to result in constitutive filamentation [3,49,50]. Both strains are able to filament in the presence of MB, while GTP-Ras1 levels are decreased (Fig 4C). Filamentation and wrinkled colony formation of these strains is not as strong as under control conditions. However, overall this shows that the effects of MB on ATP and GTP-Ras1 are upstream events in the control of *C*. *albicans* morphology and that low Ras1 signaling inhibits filamentation in the WT.

### Impact of respiratory inhibition on cellular metabolism suggests increased AMP kinase activity, but decreased Ras1 signaling is independent of AMP kinase

**Fig 5:**
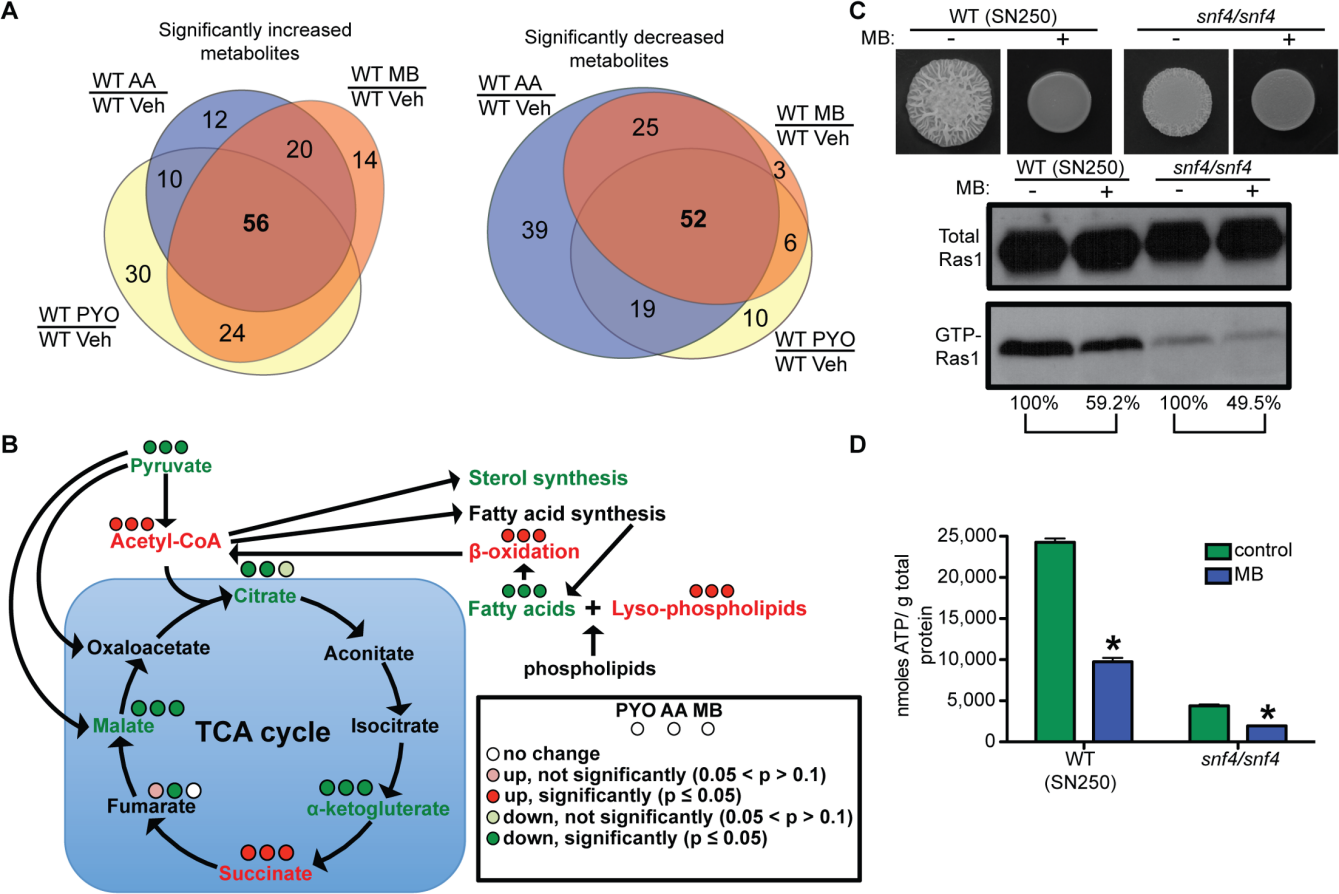
Effects of respiratory inhibitors on cellular metabolism indicate increased AMP kinase activity, but decreased Ras1 signaling is independent of AMP kinase. **(A)** Venn diagrams of significantly altered metabolites of cells treated with MB, PYO, or AA (S1 Table) showed that the majority of metabolites are changed similarly in cells treated with different respiratory inhibitors. Cells were grown for 24 h on YNBAGNP at 37°C. **(B)** Significant changes in pyruvate catabolism, sterol, fatty acid, and lyso-phospholipid levels in cells treated with MB, PYO, AA. **(C)** WT (SN250) and *snf4*/*snf4*, lacking a functional AMP kinase, grown for 24 h on YNBAGNP at 37°C (see S1A Fig panels 19 to 22) and analyzed for total and GTP-bound Ras1 levels and **(D)** intracellular ATP. (B) Percent of the GTP-Ras1/total Ras1 ratio compared to control conditions is shown. (D) Mean ± SD are shown. *p<0.05.

While MB, PYO, and AA all modulate mitochondrial activity and reduced relative GTP-Ras1 levels (Fig 3B), these compounds are not equivalent. For example, MB did not impact growth rates, while PYO and AA were inhibitory perhaps in part due to increased ROS formation. In addition, while AA and PYO led to acidification of the medium due to increased fermentation to acetate, MB did not [29,44]. Thus, we sought to determine the strongest common signals in order to gain insight into factors that control Ras1 GTP-binding. Metabolomics analysis of the WT SC5314 grown under control conditions or with MB, PYO, or AA revealed that some compounds were only differentially regulated in one condition (Fig 5A, S5 Fig, and S1 Table). For instance, only MB-grown cells had significantly high relative levels of glycerol, an alternative fermentation product, and this is consistent with the observation that the medium pH was not altered even in the presence of this fermentation-inducing mitochondrial inhibitor (S5 Fig). A large group of metabolites showed a similar pattern in the presence of all three compounds (Fig 5A); these signatures included increased lipid catabolism and decreased lipid biosynthesis (higher levels of acetyl-CoA, increased lysophospholipids, and decreased fatty acids (palmitate, oleate, and stearate)) as well as low ergosterol and related compounds (Fig 5B and S5 Fig).

The decrease of the fatty acid palmitate is particularly interesting because it is needed for one of two lipid modifications that tether Ras1 in *C*. *albicans* to the plasma membrane [51]. Loss of palmitoylation re-localizes Ras1 largely to endomembranes and changes in Ras1 localization negatively affect Ras1 activation [51]. To test if the changes in GTP-Ras1 levels seen with MB or the other inhibitors were due to re-localization of Ras1 away from the membrane, we determined GTP-Ras1 levels in two strains carrying truncated Ras1 proteins that are no longer associated with the plasma membrane. The first strain is a *ras1*/*ras1* mutant reconstituted with a *ras1* allele missing the last 67 amino acids (*ras1Δ67*) [52] and the second is a *ras1*/*ras1* mutant reconstituted with a *ras1* allele only including the conserved N-terminal region of *RAS1* (*ras1 N-term*). Interestingly, the *ras1Δ67* strain showed lower levels of GTP-Ras1 in control conditions compared to the *ras1 N-term* or wild type strain (S6 Fig) suggesting a possible GTP-binding inhibitory domain or function activated by Ras1 cleavage [52]. However, both truncated Ras1 variants showed a wild type reduction of GTP-Ras1 levels with MB (S6 Fig). Thus, while Ras1 localization is controlled by its C-terminal lipid modifications, changes in these that might occur in the presence of MB were not responsible for altered GTP-Ras1 levels.

Interestingly, the metabolomics pattern strongly resembled the response of mammalian cells to MB [53]. Furthermore, in mammalian cells the same metabolic shift due to respiratory inhibition is mediated by AMP kinase (AMPK), an energy sensor that responds to relative ATP:AMP/ADP levels. Lipids are a rich source of ATP, and AMPK induces a lipid catabolic state when ATP levels are low [54,55]. These signatures suggest that the common signal in response to PYO, MB and AA is likely low intracellular ATP. GTP-Ras1 levels were not controlled by AMPK itself as a mutant lacking the γ-subunit of AMPK (*snf4*/*snf4*) which is essential for AMPK activity in *Saccharomyces cerevisiae* [56], still shows a reduction in GTP-Ras1 and intracellular ATP upon growth with MB (Fig 5C and 5D and see S1A Fig panels 19 to 22 for cellular morphology). Under control conditions the *snf4*/*snf4* mutant has very low levels of intracellular ATP and is unable to filament (Fig 5C and S1A Fig panels 29).

### The level of Ras1 signaling depends on total intracellular ATP

**Fig 6:**
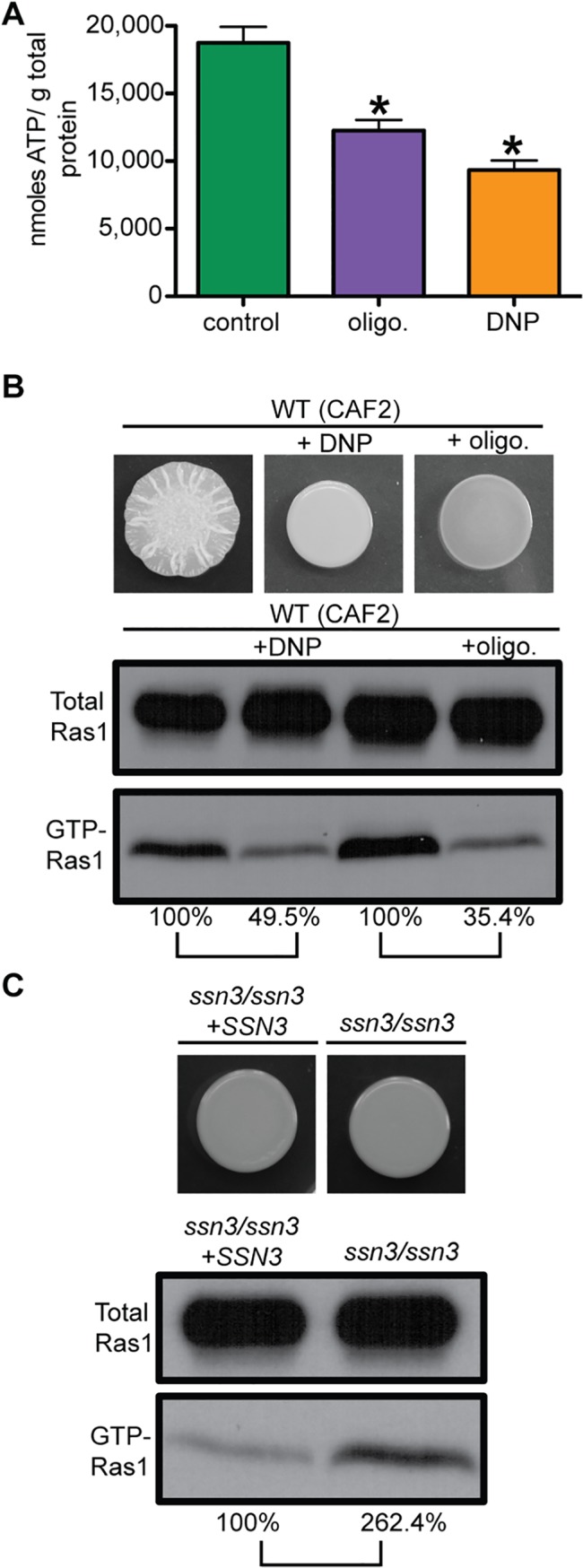
Ras1 signaling depends on total intracellular ATP levels. **(A)** Intracellular ATP levels were measured in WT (CAF2) cells exposed to dinitrophenol (DNP), oligomycin (olig.) or vehicle (control). Mean ± SD are shown. *p<0.05. Cells were grown for 24 h on YNBAGNP at 37°C. **(B)** Colony images and total Ras1 and GTP-Ras1 western blot analysis of WT (CAF2) grown for 24 h on YNBAGNP at 37°C. **(C)** Western blot analysis and colony images of the *ssn3*/*ssn3* mutant compared to its reconstituted strain grown on YNBAGP at 30°C (yeast growth) for 24 h. (B) and (C) Percent of the GTP-Ras1/total Ras1 ratio compared to control conditions is shown.

Across eukaryotes, it has been shown that diverse cellular processes from proteome function to neurotransmitter responses are directly regulated by ATP levels. Thus, phenazine-mediated repression of *C*. *albicans* filamentation may occur through effects on ATP levels, as ATP is the precursor to cAMP, a second messenger that is a key positive regulator of hyphal growth (Fig 1C) [6]. PYO reduces levels of both cAMP and its precursor ATP in human epithelial cells due to its effects on respiration and oxidative phosphorylation [57]. To determine more directly if decreased ATP levels were impacting Ras1 signaling, we examined the effects of inhibitors of the proton gradient (dinitrophenol (DNP)) and the ATP synthase (oligomycin) which each caused a significant decrease of intracellular ATP (Fig 6A). For both, relative levels of GTP-Ras1 were decreased and filamentation was repressed (Fig 6B) strongly suggesting that ATP levels were the connecting signal between mitochondrial activity and Ras1 signaling. To further test this hypothesis, we measured GTP-Ras1 levels in the *ssn3/ssn3* mutant that had been previously shown to have increased intracellular ATP due to increased oxidative metabolism, without increased growth [44], and found increased GTP-Ras1 levels compared to the reconstituted strain (Fig 6C). In summary, our data show that GTP-Ras1 levels correlate with intracellular levels of ATP.

### Decreased Ras1 signaling by methylene blue depends on the GAP Ira2 and adenylate cyclase Cyr1

**Fig 7:**
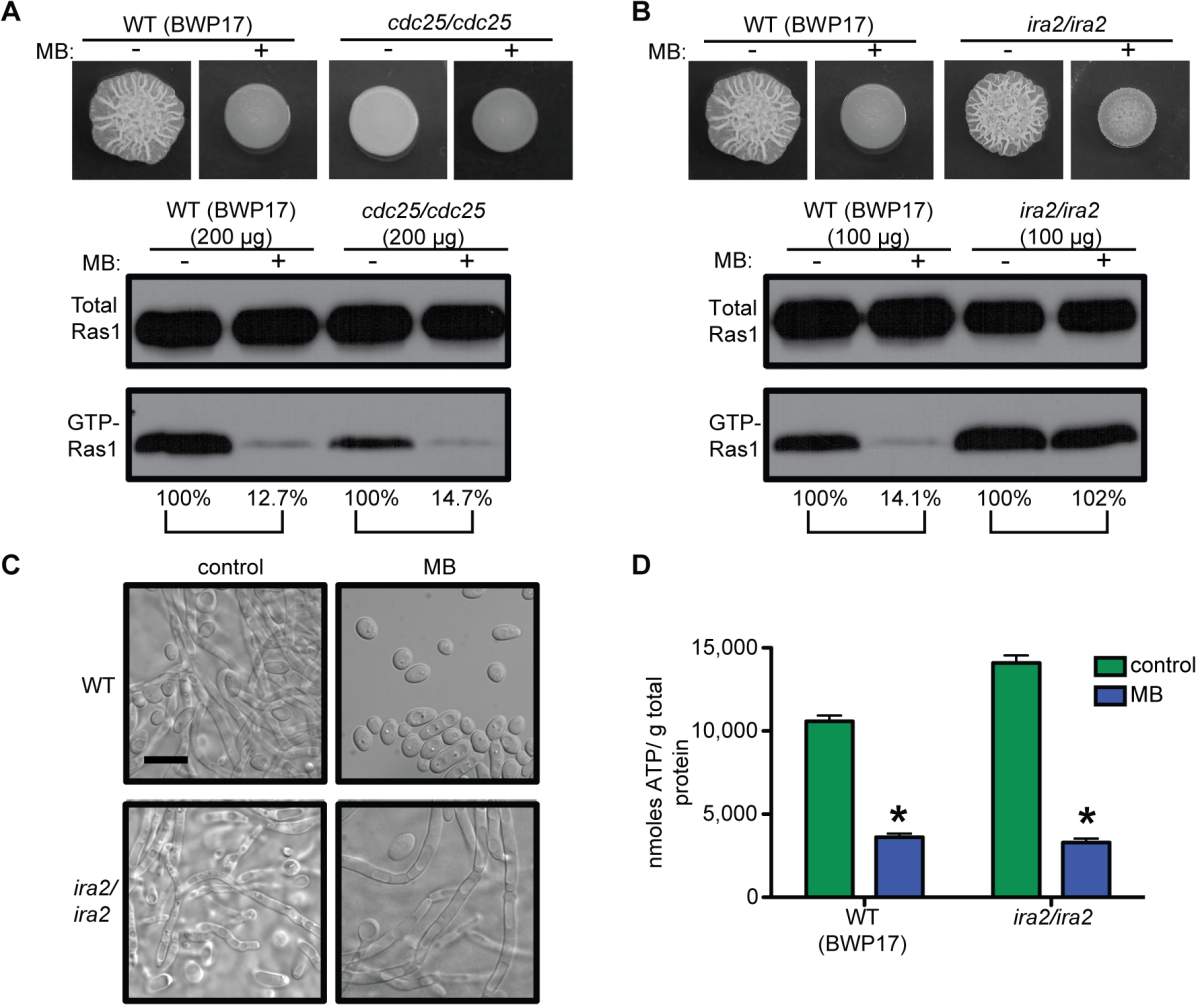
GTP-Ras1 decrease by MB is independent of the GEF Cdc25, but depends on the GAP Ira2. **(A)** and **(B)** Colony morphology and western blot analysis of the *cdc25*/*cdc25* and *ira2*/*ira2* strains compared to WT (BWP17). **(C)** Cellular morphology of the *ira2*/*ira2* strain. See S1A Fig panels 23 and 24 for cellular morphology of *cdc25*/*cdc25*. Scale bar = 10 μm. **(D)** Intracellular ATP measurement of the *ira2*/*ira2* strain compared to the WT (BWP17). Mean ± SD are shown. *p<0.05. (A) and (B) Percent of the GTP-Ras1/total Ras1 ratio compared to control conditions is reported. (A) to (D) Cells were grown for 24 h on YNBAGNP with and without MB at 37°C.

*C*. *albicans* Ras1 GTP-binding has been genetically shown to be controlled by a GEF, Cdc25, and a GAP, Ira2 [58,59]. The *cdc25*/*cdc25* mutant had low levels of GTP-Ras1, and was unable to filament, but MB caused a further reduction in GTP-Ras1 comparable to WT (Fig 7A and see S1A Fig panels 23 and 24 for cellular morphology). In contrast, loss of Ira2 resulted in a hyperfilamentous phenotype and strongly increased levels of GTP-Ras1 which were unaffected by addition of MB (Fig 7B and 7C), while ATP levels were decreased comparable to WT (Fig 7D), showing that the decrease of GTP-Ras1 by MB is Ira2 dependent.

In *S*. *cerevisiae*, Ira2 activity is negatively regulated through direct interactions with Tfs1 and positively regulated through protein stabilization by Gpb1/2 [60,61]. While *C*. *albicans tfs1/tfs1* mutants displayed phenotypes consistent with increased Ira2 activity (decreased filamentation and less GTP-Ras1), the reduction of GTP-Ras1 levels upon growth with MB was similar to that of the WT (S7A Fig). Deletion of the *C*. *albicans* Gpb1 homolog resulted in increased GTP-Ras1 levels under control conditions that were decreased with MB comparable to wild type (S7B Fig). Together the Gpb1 and Tfs1 data suggest that new inputs into Ira2 may link ATP levels to GTP-Ras1.

**Fig 8:**
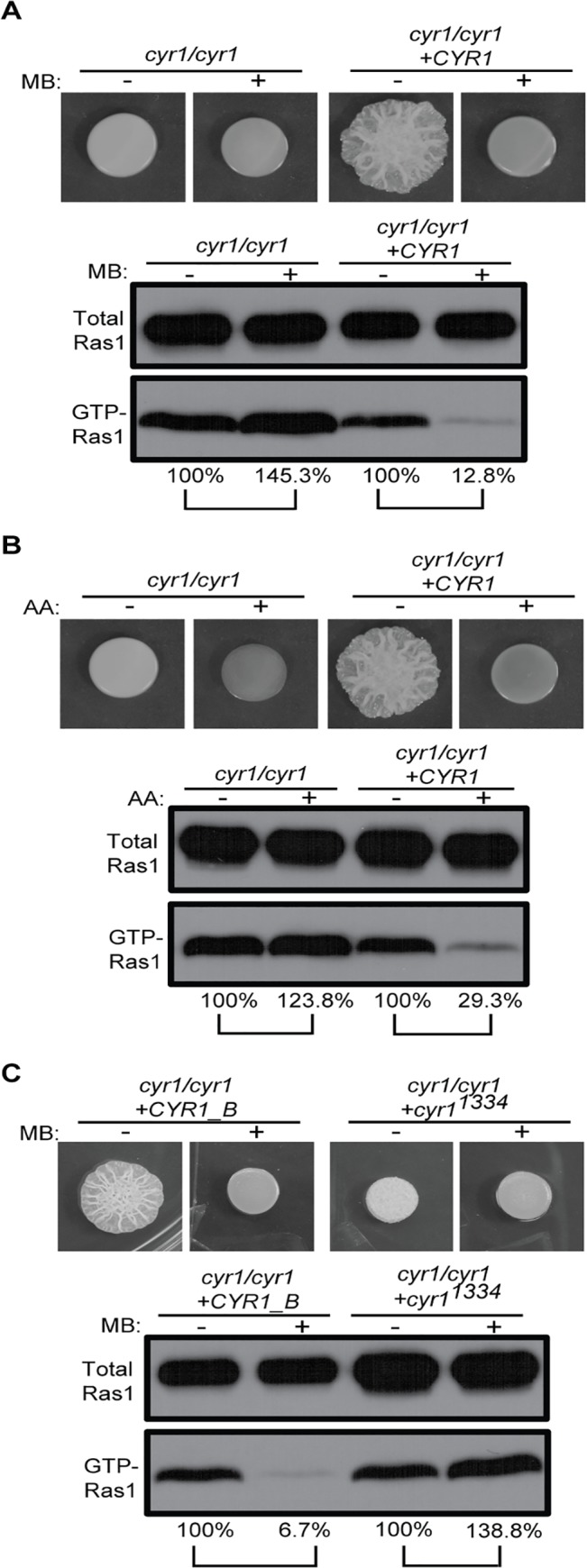
GTP-Ras1 decrease by MB depends on the adenylate cyclase Cyr1. **(A) (B) (C)** Colony morphology and western blot analysis of total Ras1 and GTP-Ras1 levels in a *cyr1*/*cyr1* mutant strain or a strain carrying a catalytically inactive *cyr1* allele (*cyr1* ^*1334*^) with MB or AA compared to their respective reconstituted strain are shown. Percent of the GTP-Ras1/total Ras1 ratio compared to control conditions is reported. Cells were grown for 24 h on YNBAGNP at 37°C. See S1A Fig panels 25 to 32 for cellular morphology.

We suspected this link may be the adenylate cyclase Cyr1, which is known to be activated by Ras1, and integrates diverse signals. The *cyr1*/*cyr1* mutant, like a *ras1*/*ras1* strain, forms smooth colonies consisting only of yeast (Fig 8 and see S1A Fig panels 25 to 28 for cellular morphology). Surprisingly, the *cyr1*/*cyr1* strain had a higher proportion of GTP-Ras1, and this increase was complemented by addition of the native *CYR1* gene. Furthermore, in the absence of Cyr1, Ras1 GTP-binding was not decreased by MB or AA but rather increased (Fig 8A and 8B). The cAMP signal itself appeared to be important, as a strain expressing only a catalytically-inactive Cyr1 (*cyr1*/*cyr1* +*cyr1* ^*1334*^) also had higher basal GTP-Ras1 levels that were increased and not decreased by MB (Fig 8C and see S1A Fig panels 29 to 32 for cellular morphology). However, neither subunit of PKA, the only known cAMP sensor, was required for the control of GTP-Ras1 levels (S7C Fig).

In summary, our data suggest that low ATP causes Cyr1-mediated activation of Ira2 activity to reduce GTP-Ras1 levels. Thus, it appears that Ras1 and Cyr1 participate in a regulatory circuit that integrates multiple signals before triggering the expression of virulence related attributes.

## Discussion

**Fig 9:**
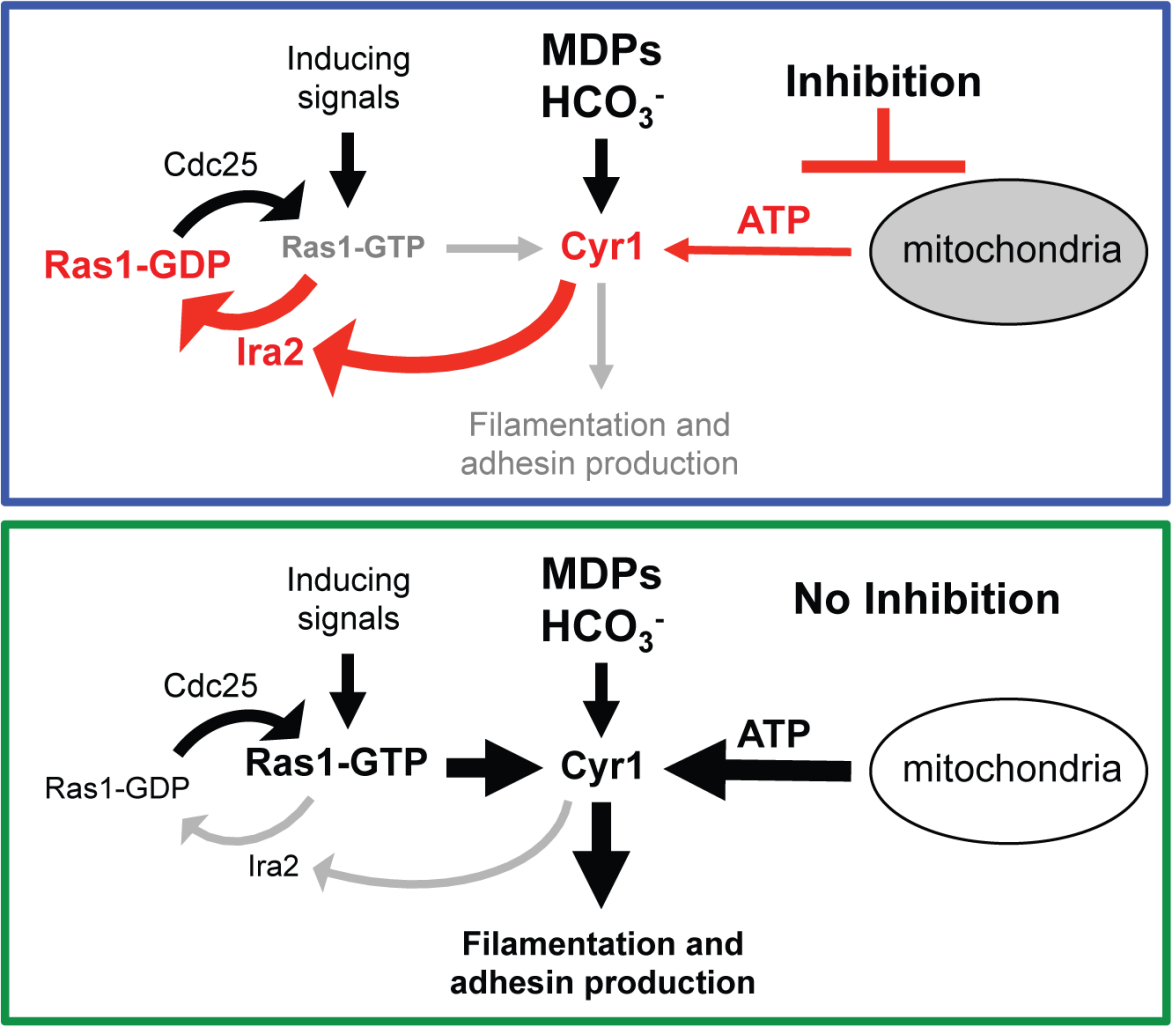
New model of the Ras1-Cyr1 signaling pathway. In the presence of MB or other respiratory inhibitors, cellular ATP levels are low. Under these conditions, GTP-bound Ras1 is rapidly turned over to GDP-bound Ras1 in a Cyr1-Ira2 dependent manner repressing the input of filamentation inducing signals and hyphal formation does not occur (upper panel). Under control conditions (no inhibition) ATP levels are high due to efficient oxidative metabolism and slow growth. In the presence of inducing signals GTP-bound Ras1 can accumulate probably because Cyr1 does not stabilize the interaction of Ras1 with Ira2. GTP-Ras1 can now effectively bind to and activate Cyr1 resulting in filamentation (lower panel). MDPs: muramyl dipeptides; HCO_3_^¯^: bicarbonate.

In this study we identified a previously unknown link between total intracellular ATP levels and Ras1 signaling in *C*. *albicans* by characterizing the mechanism by which MB inhibits the *C*. *albicans* yeast-to-hypha switch (Fig 9). Interestingly, a recent study in the yeast *S*. *cerevisiae* found that dysfunctional mitochondria decrease cAMP-PKA signaling, adhesion production, and filamentous growth further emphasizing that the link between respiratory activity and Ras1-cAMP-PKA signaling is conserved beyond the *Candida* genus [62]. The same study also showed that the filamentous-growth-specific MAPK pathway is not involved in this signaling as this pathway retained functionality in respiratory-deficient *S*. *cerevisiae* yeast cells [62]. Furthermore, while it is not known whether Ras1 signaling is important for filamentation or virulence in *C*. *tropicalis* and *C*. *parapsilosis*, when grown on YNBAGNP media with and without MB, both *Candida* species had decreased Ras1 activation state with MB indicating that the link between respiratory activity and Ras1 signaling is conserved across *Candida* species. However, whether this decrease in Ras1 activation impacts filamentation and virulence of these fungal pathogens needs to be determined in future studies.

In liquid conditions, in which *C*. *albicans* hyphal growth is fast, increased MB concentrations were necessary to see a decrease in GTP-Ras1 levels and filamentation. This requirement for higher levels of MB may be due to higher or altered respiratory activity, or differences in ATP homeostasis under well-mixed planktonic conditions. Interestingly, in our assays, GTP-bound Ras1 was lower and filamentation was inhibited by MB on both solid YPD + 5% serum medium and solid and liquid YNBAGNP. However, in liquid YPD + 5% serum conditions, the effect of MB on GTP-Ras1 levels was minor and no impact on morphology at concentrations that were not inhibitory. A recent publication by O’Meara and colleagues reported a global analysis of *C*. *albicans* morphology which showed that the role of different pathways in filamentation varied depending on the medium condition [63], and we speculate that the effects of MB on ATP pools, Ras1, and filamentation also varies in different media in ways that we cannot yet understand. Together, this variability shows the importance of understanding the interactions between nutrient sources and growth substrate and signaling inputs and outputs.

The observation that MB had no impact on Ras1 GTP-binding under yeast growth conditions in *C*. *albicans* which could indicate that intracellular ATP pools serve as a “check point” in Ras1 signaling under hypha-inducing conditions. The differential effects of MB may be related to temperature (yeast are grown at 30°C while hyphae are grown at 37°C) though MB did modulate ATP levels at 30°C. Indeed, isolated mitochondria from *C*. *albicans* were shown to be more active at 37°C compared to 30°C [34]. At lower temperatures, mammalian mitochondria have a reduced respiratory rate and hyperpolarization of the mitochondrial membrane, potentially due to decreased ATPase activity [64–67], which results in the accumulation of reduced flavins and cytochromes (Fig 3A). This state may render cells less susceptible to the action of MB. Increased respiration upon growth at 37°C most likely result in an increase in intracellular ATP concentrations which may promote or permit the hyphal growth program. MB inhibits the accumulation of ATP (Fig 3D) causing cells to stay in the yeast morphology in conditions that would normally induce filamentation.

Mutants defective in complex I and complex IV never establish a high intracellular ATP state, and are unable to undergo the yeast-to-hypha switch. Interestingly, in the presence of MB the *ndh51*/*ndh51* and *cox4*/*cox4* mutants showed an increase of GTP-Ras1 levels instead of a decrease (Fig 3C). This is consistent with observations made in mammalian cells where it has been shown that MB can restore some electron flow to dysfunctional mitochondria [43]. There was only a small but measurable increase of ATP levels in these mutants with MB which could result in a small increase in GTP-Ras1 (Fig 3D) indicating some increased electron flow might occur in the presence of MB in these *C*. *albicans* mitochondria similar to mammalian cells.

ATP production via respiration by the mitochondria is the main source of the chemical energy that fuels many different processes and pathways in the cell. Consequently, inhibition of respiration and ATP production will have a major impact on many aspects of cellular metabolism as shown by the metabolomics analysis in this study (Fig 3A, S1 Table, and S5 Fig). However, the strongly reduced ATP levels observed in the presence of MB did not block filamentous growth in the constitutively filamentous *tup1*/*tup1* mutant or *UME6*-*OE* strain suggesting that MB is not inhibiting other parallel signaling or metabolic pathways important for induction or maintenance of filamentation (Fig 4C). Filamentation and wrinkled colony formation of these strains is not as robust as under control conditions, possibly due to reduction in other Ras1-controlled pathways or other effects of low ATP levels on growth dynamics that could be Ras1 independent, but hyphal growth is clearly evident in the strains even when MB is present. Furthermore, the *NRG1-OE* strain showed that filamentation is not required for higher ATP and elevated GTP-Ras1 under filamentation inducing conditions or the effects of MB on Ras1 signaling (Fig 4A and 4B). Under the conditions tested, the *NRG1-OE* strain formed some wrinkles, however, it does not form true hyphae. Microscopy of the cells showed mainly budding yeast with some elongated yeast cells and short pseudohyphae (S1A Fig panel 21). Interestingly, the occurrence of elongated yeast cells and pseudohyphae is inhibited by MB. Previous publications with the *NRG1-OE* strain looked at YPD +serum, which in our hands is not an as strong an inducer of filamentation and wrinkle formation as YNBAGNP on plates and the overexpression level of the *NRG1*-*OE* strain might just not be enough to overcome this stronger induction completely (Fig 1A and S1B Fig) [3,42]. Wrinkle formation of *C*. *albicans* colonies by MB and other respiratory inhibitors may support the model in which wrinkles promote usage of and demand for oxygen and thus the regulation of wrinkle production is downregulated upon respiratory inhibition [68]. In the bacterium *Pseudomonas aeruginosa* wrinkled colony morphology has been shown to be a redox-driven adaptation that maximizes oxygen accessibility and increased oxygen is able to inhibit wrinkle formation [69].

It is very interesting that the response of *C*. *albicans* to respiratory inhibition is similar to what has been seen in mammalian cells [53]. We observed a metabolome profile typical for AMP kinase activation, which has also been shown in mammalian cells exposed to MB [70]. This activation is not surprising as AMP kinase is a known energy sensor that measures ratios of ATP to ADP/AMP [55] that is activated when cellular energy status is low in eukaryotes. To increase ATP availability, the cells increase the catabolism of energy stores, such as fatty acids, and the biosynthesis of “costly” fatty acids and ergosterol is decreased (S1 Table, S5 Fig) [54,55,71]. Our data indicate that AMPK is needed to sustain levels of ATP, as intracellular ATP levels were very low in the *snf4*/*snf4* strain (Fig 5D). We suspect this is the reason why GTP-Ras1 levels are so low and why this strain does not form filaments (Fig 5C). In agreement, a recent study showed that loss of only the kinase activity due to a point mutation in Snf1, an essential protein in *C*. *albicans*, inhibited the yeast-to-hyphal switch indicating how important AMPK activity is for energy homeostasis and filamentation [72]. Even though GTP-Ras1 and ATP levels are low in the *snf4*/*snf4* strain, they are still responsive to MB showing that AMPK is not necessary for this signaling pathway. Overall, we believe that by understanding how *C*. *albicans* metabolism changes in different environments, we can use this fungus as an important probe for conditions within the host in states of health and disease.

In many cells, ATP concentrations control diverse processes such as autophagy [39], the retrograde response pathway [73], activation of neutrophils [74], and neurotransmitter responses [75]. ATP can act as an essential co-factor or can be recognized directly by binding to a receptor which triggers signaling. We do not yet know how ATP levels impact the Ras1 signaling cascade. One candidate ATP sensor is adenylate cyclase, Cyr1, itself. Cyr1 catalyzes the conversion of ATP to cAMP and thus has an ATP binding site that could function as a sensor of ATP levels. In *S*. *cerevisiae*, studies indicate that Cyr1 acts as a scaffold protein for Ras2 (homolog to Ras1 in *C*. *albicans*) interactions with Ira2 [76] and one could imagine that this scaffold activity may be regulated by ATP concentration. We know that Cyr1 signaling is required for the effects of MB in *C*. *albicans* as a catalytically inactive Cyr1, which can still serve some structural roles, was also insensitive to the Ras1-inhibiting effects of MB (Fig 8C). Indeed the *cyr1*/*cyr1* mutant and the strain expressing catalytically inactive Cyr1 showed an increase of GTP-Ras1 with MB. Together with previously published data showing that Ras1 signaling is important for mitochondrial activity in *S*. *cerevisiae*, this might indicate that the mitochondria are not functioning normally in these strains and that like in mammalian cells and the *cox4/cox4* and *ndh51/ndh51* strains MB is able restore some electron flow to these dysfunctional mitochondria [43,77].

In *S*. *cerevisiae*, Ira2 has been shown to interact with the protein kinase A regulatory subunit, and Cyr1 and Ira2 have both been found at the plasma membrane and on mitochondria [78] providing further support for the potential for interactions between Cyr1 and Ira2, probably in ways that respond to mitochondrial activity [79] (Fig 9). These reports, with the data presented here, suggest that Cyr1 and Ras1 form a master regulatory circuit. Furthermore, the canonical Ras1 signaling pathway model has to be restructured from a pathway in which Cyr1 is just a factor downstream of Ras1 that is activated by GTP-bound Ras1 (Fig 1C) to a new model in which Cyr1 and Ras1 influence each other and together with Ira2 form a master-regulatory network necessary to coordinate the response to different environmental and intracellular signals in order to decide the fate of the cell (Fig 9).

These data reveal important aspects of the regulatory cascade that controls the *C*. *albicans* switch to a state more capable of causing host damage (Fig 9). Our findings indicate that the energy status of the cell is one of the most important signals involved in the decision of *C*. *albicans* to undergo the yeast-to-hyphae switch as it is able to override an array of filamentation inducing signals (Fig 9) Thus, host or host microbiome factors that impact energy levels will likely modulate *C*. *albicans* Ras1 signaling. Previous studies showed that Cyr1 can be directly activated by bicarbonate and muramyl dipeptides (MDPs) (Fig 9), though MDPs are only weak activators of filamentation in the absence of Ras1 [80,81] showing that Ras1 input is required for strong MDP-induced filamentation. Indeed, clinical data have linked the use of antibacterials to increased risk of *C*. *albicans* infections in multiple distinct body sites with very different bacterial community compositions [7–14]. In addition, numerous studies have shown that many bacteria inhibit *C*. *albicans* filamentation [13,14]. Future studies will focus on establishing whether repression in Ras1 activation, through modulation of ATP by competition with other microbes, contributes to the control of *Candida* behavior in a healthy mucosal microbial community. Furthermore, the future will show if known therapies or strategies can be used to favor benign host-*Candida* interactions by promoting low Ras1 activity.

## Materials and Methods

### Strains and growth conditions

All *C*. *albicans* strains were streaked from-80°C onto YPD (1% yeast extract, 2% peptone, 2% glucose) plates every 8–10 days and maintained at room temperature. All strains used in this study can be found in S2 Table. Overnight cultures were grown in 5 ml of YPD, supplemented with uridine as necessary, and washed in distilled water (dH_2_O) prior to use. *C*. *albicans* cells were mostly grown under filament-inducing conditions which included 37°C on YNBAGNP (1.5% agar, 0.67% yeast nitrogen base medium with ammonium sulfate (RPI Corp), 10 mM dextrose, 5 mM N-acetylglucosamine (GlcNAc), and 2% [wt/vol] casamino acids (BD Bacto), 25 mM potassium phosphate buffer). Cells were also grown on YPD + 5% fetal bovine serum when indicated. For yeast growth conditions *C*. *albicans* cells were grown at 30°C on YNBGP (1.5% agar, 0.67% yeast nitrogen base medium with ammonium sulfate (RPI Corp), 10 mM dextrose, 25 mM potassium phosphate buffer). For filamentation-inducing liquid growth conditions media were prepared as described above without the addition of agar and cells were incubated in the roller drum for 12 hours at 37°C.

Stock solutions were prepared of: methylene blue (MB) (Fisher Scientific)- 3 mM in dH_2_O; pyocyanin (PYO) (Cayman Chemicals)- 30 mM in 100% ethanol (EtOH); Antimycin A (AA) (Sigma)- 10 mM in 100% EtOH; oligomycin (Sigma) – 8 mg/ml in 100% EtOH; Dinitrophenol (DNP) (Sigma) – 100 mM in DMSO; menadione (Sigma) – 50 mM in 100% EtOH. All experiments were conducted in the dark to avoid light-induced ROS production.

### Strain construction

The deletion mutant strains were constructed in the BWP17 strain background using a previously described method [58,82]. Briefly, gene-disruption cassettes for transformation were amplified using ~75 bp primers and the plasmids, pRS-*ARG4* or pGEM-*HIS1* [82] which contain *ARG4* and *HIS1* for PCR-directed integration. The forward primer was designed to have homology to 50 bp sequence upstream of the gene of interest start codon while the reverse primer had homology to the 50 bp sequence following the stop-codon. Both the primers were flanked by a 20 bp sequence homologous to the plasmids, as mentioned above. Sequential transformations of these gene-disruption cassettes into *C*. *albicans* BWP17 strain yielded the deletion strain. Plasmid pSM2 and pSMTC were used to complement the *cyr1*/*cyr1* strain only with *URA3* or with *CYR1*-*URA3* at the *URA3* locus [80]. The *ira2*/*ira2* strain was reconstituted with *URA3* using the pClp10 plasmid at the *RP10* locus [83]. Strain *ras1*/*ras1* + *ras1* N-term was generated by transforming DH482 with PacI linearized pAP13+ras1 N-term and integration at the endogenous RAS1 locus was confirmed by PCR. To construct pAP13+ras1 N-term a PCR product encoding the first 161 residues of Ras1 was amplified from pAP14 [51] with primers RAS1XhoIF [51] and Ras1delta129BamHI-R, digested with XhoI and BamHI and ligated into similarly digested pAP13. All plasmids and primers used in this study can be found in S3 and S4 Tables.

### Macroscopic and microscopic analysis of cells in colonies

For wrinkled colony formation, 10 μl from overnight cultures re-suspended in dH_2_O at an optical density (OD) of 8.0 were spotted onto YNBAGNP unless otherwise specified. The medium was supplemented with methylene blue (MB) from a 3 mM stock solution to a final concentration of 1.5 μM. 5 μM MB was used for the metabolomics experiment. Pyocyanin (PYO), Antimycin A (AA), and oligomycin (olig.) were added to the medium to a final concentration of 20 μM, 2.5 μM, and 7.5 μg/ml, respectively, or an equivalent volume of 100% ethanol (vehicle). Dinitrophenol (DNP) was added to the medium to a final concentration of 2 mM and menadione was added to the medium to a final concentration of 0.125 mM or an equivalent volume of vehicle solution. Cells were incubated at 37°C for 25 h.

Colonies were imaged after 24 h with a digital camera. Unless otherwise noted, all spot assays were completed as at least three independent replicates and a representative data set is shown. Cell morphology in colonies was assessed using a ZeissAxiovert inverted microscope equipped with a 100x long working distance objective and Axiovision software. To image the morphology of cells within the colony, the cells were resuspended in water, then applied to an agarose-coated slide to immobilize cells of different morphologies. The images shown were representative of the make up of the entire colony.

### Active Ras1 pull-down and immunoblotting

For western blot analysis spot colonies were scraped from agar plates after 24 h incubation at the conditions indicated, washed into a collection tube with dH_2_O and, after centrifugation, immediately snap-frozen in an ethanol/dry ice bath. Lysate preparation was conducted as previously published, with some modifications [51]. Whole-cell lysates were prepared by resuspending cells in Lysis/Binding/Wash Buffer (Active Ras Pull-Down and Detection Kit, Pierce) with protease inhibitors (Halt Protease Inhibitor Single-Use Cocktail, Pierce) and disrupting cells with glass beads in a Bio-Spec bead beater with six rounds of 50 seconds disruptions at 4°C and 1 minute rests on ice. Protein concentrations were determined by Bradford assay (BioRad).

Active or GTP-bound Ras1 was isolated utilizing the Active Ras Pull-Down and Detection Kit (Pierce) following the manufacturer’s instructions. In general, 200 μg of total protein were used for the pull-down unless otherwise specified. Due to the strong increase of GTP-Ras1 levels in *ira2*/*ira2* strain only 100 μg of total protein were used (indicated in the figure). 12.5 μl of the pull-down samples containing active Ras1, and, for the input control, a total of 10 μg total protein diluted in SDS loading buffer were separated by SDS-PAGE, transferred to polyvinylidene difluoride (PVDF) with the Trans-Blot Turbo Transfer system (BioRad), and detected with monoclonal anti-Ras clone 10 (1.5 μg/ml; Millipore), followed by secondary detection with goat anti-mouse (Pierce) and enhanced chemiluminescent visualization (Pierce). As a control protein Pma1 was detected as described previously [52]. Densitometry analysis of Ras1 levels on Western blots was conducted with ImageJ [84].

### Gene expression

Nanostring nCounter (Nanostring Technologies) analysis was used to quantify *C*. *albicans* gene expression. After 24 h spot colonies were harvested and fungal RNA was isolated using MasterPure Yeast RNA Purification Kit (Epicentre). Each Nanostring reaction mixture contained 80 ng fungal RNA, hybridization buffer, reporter and capture probes. Overnight hybridization of RNA with probes at 65°C preceded sample preparation using Nanostring prep station. Targets were counted on the nCounter using 255 fields of view per sample [85]. Raw counts for hyphal and yeast specific transcripts (*HWP1*, *ECE1*, *HGC1*, *HYR1*, *ALS3*, *YWP1* and *ALS4*) were normalized within each sample to the geometric mean of two *C*. *albicans* housekeeping genes (*ACT1*, *PMA1*) and scaled to WT control conditions; the numerical average was taken from three biological replicates. Heat maps were developed using Z-scoring of Nanostring counts of selected yeast- and hyphal-specific genes using the “heatmap.2” function in the “gplots” package [86] in R (R Foundation for Statistical Computing, Vienna, Austria).

### Metabolomics

Spot assays were completed as previously described on YNBGNP agar plates and incubated at 37°C for 24 h. Cells were harvested, by scraping colonies from the surface of the agar using a coverslip, and then snap-frozen in an ethanol/dry ice bath. A total of 5 biological replicates were submitted to Metabolon for metabolite profiling, by GC/MS and LC/MS, of SC5314 wild type treated with vehicle (EtOH), 5 μM MB, 20 μM PYO, or 2.5 μM AA. All metabolites with mean values that had significant differences (p≤0.05) between treated and untreated samples were clustered into the 2 groups “UP” (upregulated ≥1.00-fold) or “DOWN” (downregulated <1.00-fold). VennMaster (http://sysbio.uni-ulm.de/?Software:VennMaster) [87] was used to determine the overlap of biochemicals that were either “UP” or “DOWN” in any of the treated samples. To visualize the result of this overlap analysis the tool euler*APE* (http://www.eulerdiagrams.org/eulerAPE) [88] was used to generate the Euler diagrams.

### ATP quantification

Spot assays were completed as previously described on YNBGNP agar plates with and without 1.5 μM MB or 7.5 μg/ml oligomycin and incubated at 37°C for 24 h. After harvesting by scraping colonies from the surface of the agar using a coverslip, the cells were disrupted with glass beads and 1x PBS in a Bio-Spec bead beater with 3 rounds of 60 seconds disruptions at 4°C and 1 minute rests on ice in between. A standard curve was prepared using Adenosine 5’-triphosphate disodium salt hydrate (Sigma). ATP levels were measured using the CellTiter-Glo Luminescent Cell Viability Assay (Promega) following the manufacturer’s instructions. The luminescent signal, which is proportional to ATP levels, was measured using a Tecan Infinite 200 Pro equipped with Magellan software (Tecan). All data were normalized to the protein concentration of each sample, which was determined using a Bradford Assay (BioRad). Three independent biological replicates, each including three technical replicates, were conducted and a representative data set is presented.

### Gene ID—CGD systematic name

*RAS1*: C2_10210C_A; *CYR1*: C7_03070C_A; *TPK1*: C1_10220C_A; *TPK2*: C2_07210C_A; *CDC25*: C3_03890W; *IRA2*: C1_12450C_A; *TFS1*: C5_00930C_A; *GPB1*: C4_02150C_A; *NDH51*: C2_04550C_A; *SDH1*: C1_05260C_A; *AOX1-A*: C1_09160W_A; *AOX1-B*: C1_09150W_A; *COX4*: C2_01620W_A; *SNF4*: C6_03920W_A; *NRG1*: C7_04230W_A; *UME6*: C1_06280C_A; *EFG1*: CR_07890W_A; *TUP1*: C1_00060W_A; *SSN3*: C2_04260W_A

## Supporting Information

S1 Fig MB effects on cell morphology. Cells were grown for 24 h on **(A)** YNBAGNP or **(B)** YPD +5% serum at 37°C in spot colonies before imaging. Microscopy pictures of CAF2 in (A) are identical to images shown in Fig 4C. Scale bar = 10 μm.
(TIF)

S2 Fig MB effects on Ras1 signaling also occur in liquid media and on YPD, and are not due to growth inhibition. **(A)** Microscopy images and Western blot analysis of WT (CAF2) grown in liquid YNBAGNP media for 12 hours at 37°C. Scale bar = 100 μm. **(B)** Growth of *C*. *albicans* strain *NRG1-OE* in the presence of different concentrations of MB. Data represents the mean and SD of three biological replicates grown in liquid YNBAGNP at 37°C. **(C)** Smooth colony morphology (consisting of yeast only) in the presence and absence of MB at yeast growth conditions (30°C; YNBGP; 24 h). **(D)** WT (CAF2) grown on YPD +5% serum with and without MB (see S1B Fig for cellular morphology). Western blot analysis of total Ras1 and GTP-Ras1 levels shown. Cells were grown for 24 h at 37°C (E) Microscopy images and Western blot analysis of WT (CAF2) grown in liquid YPD +5% serum media for 12 hours at 37°C. Scale bar = 100 μm. (A), (D), and (E) Percent of the GTP-Ras1/total Ras1 ratio compared to WT control conditions is shown.
(TIF)

S3 Fig Loss of mitochondrial ETC complex II or the alternative oxidase has no effect on Ras1 GTP-binding or the decrease of GTP-Ras1 levels by MB (A) (B) Colony morphology and western blot analysis of the WT (CAF2), *sdh1*/*sdh1* (complex II), and *aox1*/*aox1 aox2*/*aox2* (alternative oxidase) strains grown on YNBAGNP for 24 h at 37°C with and without MB are shown. (B) Western samples were run on the same gel. Percent of the GTP-Ras1/total Ras1 ratio compared to WT control conditions is shown.
(TIF)

S4 Fig MB effects on Ras1 signaling in an *efg1*/*efg1* mutant. **(A)** Western blot analysis and **(B)** intracellular ATP measurements comparing WT (CAF2) with the yeast locked *efg1*/*efg1* strain are shown. Cells were grown on YNBAGNP for 24 h at 37°C with and without MB. (A) Percent of the GTP-Ras1/total Ras1 ratio compared to control conditions is reported. (B) Mean ± SD are shown. *p<0.05.
(TIF)

S5 Fig Schematic of changed metabolites in cells exposed to PYO, AA, MB compared to vehicle treated cells. Data represent a subset of metabolites changed. See S1 Table for the complete data set.
(TIF)

S6 Fig Decreased Ras1 activation by MB is independent of Ras1 localization. Colony morphology and western blot analysis of WT (CAF2), *ras1*/*ras1* +*ras1*Δ*67*, and *ras1*/*ras1* +*ras1N-term* are shown. Percent of the GTP-Ras1/total Ras1 ratio compared to WT control conditions is shown. Cells were grown on YNBAGNP for 24 h at 37°C with and without MB.
(TIF)

S7 Fig Decreased GTP-Ras1 levels are independent of the Ira2 inhibitor Tfs1, the Ira2 stabilizer Gpb1, or either of the two PKA subunits. **(A) (B) (C)** Colony morphology and western blot analysis of total Ras1 and GTP-Ras1 in the WT (BWP17, CAF2), *tfs1*/*tfs1*_9, *tfs1*/*tfs1*_21, *gpb1*/*gpb1*, *tpk1*/*tpk1*, and *tpk2*/*tpk2* strains are shown. Cells were grown on YNBAGNP for 24 h at 37°C with and without MB. (B) Western samples were run on the same gel. Percent of the GTP-Ras1/total Ras1 ratio compared to control conditions is reported. Percent of the GTP-Ras1/total Ras1 ratio compared to WT control conditions is shown.
(TIF)

S1 Table Metabolomics analysis of *C*. *albicans* with MB, AA, or PYO compared to vehicle control. (XLSX)

S2 Table Strains used in this study. (DOCX)

S3 Table Plasmids used in this study. (DOCX)

S4 Table Sequences of primers used in this study. (DOCX)

## References

[1] Pfaller MA, Diekema DJ (2010) Epidemiology of invasive mycoses in North America. Crit Rev Microbiol 36: 1–53. 10.3109/10408410903241444 20088682

[2] Casalinuovo IA, Di Francesco P, Garaci E (2004) Fluconazole resistance in *Candida albicans*: a review of mechanisms. Eur Rev Med Pharmacol Sci 8: 69–77. 15267120

[3] Peters BM, Palmer GE, Nash AK, Lilly EA, Fidel PL Jr., et al (2014) Fungal morphogenetic pathways are required for the hallmark inflammatory response during *Candida albicans* vaginitis. Infect Immun 82: 532–543. 10.1128/IAI.01417-13 24478069 PMC3911367

[4] Lo HJ, Kohler JR, DiDomenico B, Loebenberg D, Cacciapuoti A, et al (1997) Nonfilamentous *C*. *albicans* mutants are avirulent. Cell 90: 939–949. 9298905 10.1016/s0092-8674(00)80358-x

[5] Mitchell AP (1998) Dimorphism and virulence in *Candida albicans* . Curr Opin Microbiol 1: 687–692. 10066539 10.1016/s1369-5274(98)80116-1

[6] Hogan DA, Sundstrom P (2009) The Ras/cAMP/PKA signaling pathway and virulence in *Candida albicans* . Future Microbiol 4: 1263–1270. 10.2217/fmb.09.106 19995187

[7] Kashkin PN, Krassilnicov NA, Nekachalov VY (1961) *Candida* complications after antibiotic therapy. Mycopathologia 14: 173–188. 13751663 10.1007/BF02057324

[8] Samonis G, Anastassiadou H, Dassiou M, Tselentis Y, Bodey GP (1994) Effects of broad-spectrum antibiotics on colonization of gastrointestinal tracts of mice by *Candida albicans* . Antimicrob Agents Chemother 38: 602–603. 8203861 10.1128/aac.38.3.602 PMC284504

[9] Samonis G, Gikas A, Toloudis P, Maraki S, Vrentzos G, et al (1994) Prospective study of the impact of broad-spectrum antibiotics on the yeast flora of the human gut. Eur J Clin Microbiol Infect Dis 13: 665–667. 7813500 10.1007/BF01973996

[10] Krcmery V Jr., Matejicka F, Pichnova E, Jurga L, Sulcova M, et al (1999) Documented fungal infections after prophylaxis or therapy with wide spectrum antibiotics: relationship between certain fungal pathogens and particular antimicrobials? J Chemother 11: 385–390. 10632385 10.1179/joc.1999.11.5.385

[11] Xu J, Schwartz K, Bartoces M, Monsur J, Severson RK, et al (2008) Effect of antibiotics on vulvovaginal candidiasis: a MetroNet study. J Am Board Fam Med 21: 261–268. 10.3122/jabfm.2008.04.070169 18612052

[12] Lynch AS, Robertson GT (2008) Bacterial and fungal biofilm infections. Annu Rev Med 59: 415–428. 17937586 10.1146/annurev.med.59.110106.132000

[13] Peleg AY, Hogan DA, Mylonakis E (2010) Medically important bacterial-fungal interactions. Nat Rev Microbiol 8: 340–349. 10.1038/nrmicro2313 20348933

[14] Morales DK, Hogan DA (2010) *Candida albicans* interactions with bacteria in the context of human health and disease. PLoS Pathog 6: e1000886 10.1371/journal.ppat.1000886 20442787 PMC2861711

[15] Davis-Hanna A, Piispanen AE, Stateva LI, Hogan DA (2008) Farnesol and dodecanol effects on the *Candida albicans* Ras1-cAMP signalling pathway and the regulation of morphogenesis. Mol Microbiol 67: 47–62. 18078440 10.1111/j.1365-2958.2007.06013.x PMC3782305

[16] Hall RA, Turner KJ, Chaloupka J, Cottier F, De Sordi L, et al (2011) The quorum-sensing molecules farnesol/homoserine lactone and dodecanol operate via distinct modes of action in *Candida albicans* . Eukaryot Cell 10: 1034–1042. 10.1128/EC.05060-11 21666074 PMC3165441

[17] Rocha CR, Schroppel K, Harcus D, Marcil A, Dignard D, et al (2001) Signaling through adenylyl cyclase is essential for hyphal growth and virulence in the pathogenic fungus *Candida albicans* . Mol Biol Cell 12: 3631–3643. 11694594 10.1091/mbc.12.11.3631 PMC60281

[18] Leberer E, Harcus D, Dignard D, Johnson L, Ushinsky S, et al (2001) Ras links cellular morphogenesis to virulence by regulation of the MAP kinase and cAMP signalling pathways in the pathogenic fungus *Candida albicans* . Mol Microbiol 42: 673–687. 11722734 10.1046/j.1365-2958.2001.02672.x

[19] Park H, Myers CL, Sheppard DC, Phan QT, Sanchez AA, et al (2005) Role of the fungal Ras-protein kinase A pathway in governing epithelial cell interactions during oropharyngeal candidiasis. Cell Microbiol 7: 499–510. 15760450 10.1111/j.1462-5822.2004.00476.x

[20] Boguski MS, McCormick F (1993) Proteins regulating Ras and its relatives. Nature 366: 643–654. 8259209 10.1038/366643a0

[21] Feng Q, Summers E, Guo B, Fink G (1999) Ras signaling is required for serum-induced hyphal differentiation in *Candida albicans* . J Bacteriol 181: 6339–6346. 10515923 10.1128/jb.181.20.6339-6346.1999 PMC103768

[22] Fang HM, Wang Y (2006) RA domain-mediated interaction of Cdc35 with Ras1 is essential for increasing cellular cAMP level for *Candida albicans* hyphal development. Mol Microbiol 61: 484–496. 16856944 10.1111/j.1365-2958.2006.05248.x

[23] Cassola A, Parrot M, Silberstein S, Magee BB, Passeron S, et al (2004) *Candida albicans* lacking the gene encoding the regulatory subunit of protein kinase A displays a defect in hyphal formation and an altered localization of the catalytic subunit. Eukaryot Cell 3: 190–199. 14871949 10.1128/EC.3.1.190-199.2004 PMC329502

[24] Zelada A, Castilla R, Passeron S, Giasson L, Cantore ML (2002) Interactions between regulatory and catalytic subunits of the *Candida albicans* cAMP-dependent protein kinase are modulated by autophosphorylation of the regulatory subunit. Biochim Biophys Acta 1542: 73–81. 11853881 10.1016/s0167-4889(01)00168-9

[25] Mayer FL, Wilson D, Hube B (2013) *Candida albicans* pathogenicity mechanisms. Virulence 4: 119–128. 10.4161/viru.22913 23302789 PMC3654610

[26] Yi S, Sahni N, Daniels KJ, Lu KL, Srikantha T, et al (2011) Alternative mating type configurations (a/alpha versus a/a or alpha/alpha) of *Candida albicans* result in alternative biofilms regulated by different pathways. PLoS Biol 9: e1001117 10.1371/journal.pbio.1001117 21829325 PMC3149048

[27] Huang G, Yi S, Sahni N, Daniels KJ, Srikantha T, et al (2010) N-acetylglucosamine induces white to opaque switching, a mating prerequisite in *Candida albicans* . PLoS Pathog 6: e1000806 10.1371/journal.ppat.1000806 20300604 PMC2837409

[28] Kerr JR, Taylor GW, Rutman A, Hoiby N, Cole PJ, et al (1999) *Pseudomonas aeruginosa* pyocyanin and 1-hydroxyphenazine inhibit fungal growth. J Clin Pathol 52: 385–387. 10560362 10.1136/jcp.52.5.385 PMC1023078

[29] Morales DK, Grahl N, Okegbe C, Dietrich LE, Jacobs NJ, et al (2013) Control of *Candida albicans* metabolism and biofilm formation by *Pseudomonas aeruginosa* phenazines. MBio 4: e00526–00512. 10.1128/mBio.00526-12 23362320 PMC3560528

[30] Laursen JB, Nielsen J (2004) Phenazine natural products: biosynthesis, synthetic analogues, and biological activity. Chem Rev 104: 1663–1686. 15008629 10.1021/cr020473j

[31] French SW, Palmer DS, Sim WA (1973) Phenazine methosulfate uptake by rat liver mitochondria. Can J Biochem 51: 235–240. 4700341 10.1139/o73-030

[32] O'Malley YQ, Abdalla MY, McCormick ML, Reszka KJ, Denning GM, et al (2003) Subcellular localization of *Pseudomonas* pyocyanin cytotoxicity in human lung epithelial cells. Am J Physiol Lung Cell Mol Physiol 284: L420–430. 12414438 10.1152/ajplung.00316.2002

[33] O'Malley YQ, Reszka KJ, Rasmussen GT, Abdalla MY, Denning GM, et al (2003) The *Pseudomonas* secretory product pyocyanin inhibits catalase activity in human lung epithelial cells. Am J Physiol Lung Cell Mol Physiol 285: L1077–1086. 12871859 10.1152/ajplung.00198.2003

[34] Guedouari H, Gergondey R, Bourdais A, Vanparis O, Bulteau AL, et al (2014) Changes in glutathione-dependent redox status and mitochondrial energetic strategies are part of the adaptive response during the filamentation process in *Candida albicans* . Biochim Biophys Acta 1842: 1855–1869. 10.1016/j.bbadis.2014.07.006 25018088

[35] Armstrong AV, Stewart-Tull DE (1971) The site of the activity of extracellular products of *Pseudomonas aeruginosa* in the electron-transport chain in mammalian cell respiration. J Med Microbiol 4: 263–270. 4328182 10.1099/00222615-4-2-263

[36] Armstrong AV, Stewart-Tull DE, Roberts JS (1971) Characterisation of the *Pseudomonas aeruginosa* factor that inhibits mouse-liver mitochondrial respiration. J Med Microbiol 4: 249–262. 4998856 10.1099/00222615-4-2-249

[37] Stewart-Tull DE, Armstrong AV (1972) The effect of 1-hydroxyphenazine and pyocyanin from *Pseudomonas aeruginosa* on mammalian cell respiration. J Med Microbiol 5: 67–73. 4623349 10.1099/00222615-5-1-67

[38] Hamanaka RB, Chandel NS (2010) Mitochondrial reactive oxygen species regulate cellular signaling and dictate biological outcomes. Trends Biochem Sci 35: 505–513. 10.1016/j.tibs.2010.04.002 20430626 PMC2933303

[39] Tait SW, Green DR (2012) Mitochondria and cell signalling. J Cell Sci 125: 807–815. 10.1242/jcs.099234 22448037 PMC3311926

[40] Kasozi DM, Gromer S, Adler H, Zocher K, Rahlfs S, et al (2011) The bacterial redox signaller pyocyanin as an antiplasmodial agent: comparisons with its thioanalog methylene blue. Redox Rep 16: 154–165. 10.1179/174329211X13049558293678 21888766 PMC6837537

[41] Schirmer RH, Adler H, Pickhardt M, Mandelkow E (2011) "Lest we forget you—methylene blue…". Neurobiol Aging 32: 2325 e2327–2316. 10.1016/j.neurobiolaging.2010.12.012 21316815

[42] Saville SP, Lazzell AL, Monteagudo C, Lopez-Ribot JL (2003) Engineered control of cell morphology in vivo reveals distinct roles for yeast and filamentous forms of *Candida albicans* during infection. Eukaryot Cell 2: 1053–1060. 14555488 10.1128/EC.2.5.1053-1060.2003 PMC219382

[43] Lee KK, Boelsterli UA (2014) Bypassing the compromised mitochondrial electron transport with methylene blue alleviates efavirenz/isoniazid-induced oxidant stress and mitochondria-mediated cell death in mouse hepatocytes. Redox Biol 2C: 599–609. 10.1016/j.redox.2014.03.003 25460728 PMC4297936

[44] Lindsay AK, Morales DK, Liu Z, Grahl N, Zhang A, et al (2014) Analysis of *Candida albicans* mutants defective in the Cdk8 module of mediator reveal links between metabolism and biofilm formation. PLoS Genet 10: e1004567 10.1371/journal.pgen.1004567 25275466 PMC4183431

[45] McDonough JA, Bhattacherjee V, Sadlon T, Hostetter MK (2002) Involvement of *Candida albicans* NADH dehydrogenase complex I in filamentation. Fungal Genet Biol 36: 117–127. 12081465 10.1016/S1087-1845(02)00007-5

[46] Stoldt VR, Sonneborn A, Leuker CE, Ernst JF (1997) Efg1p, an essential regulator of morphogenesis of the human pathogen *Candida albicans*, is a member of a conserved class of bHLH proteins regulating morphogenetic processes in fungi. EMBO J 16: 1982–1991. 9155024 10.1093/emboj/16.8.1982 PMC1169801

[47] Bockmuhl DP, Ernst JF (2001) A potential phosphorylation site for an A-type kinase in the Efg1 regulator protein contributes to hyphal morphogenesis of *Candida albicans* . Genetics 157: 1523–1530. 11290709 10.1093/genetics/157.4.1523 PMC1461612

[48] Sonneborn A, Bockmuhl DP, Gerads M, Kurpanek K, Sanglard D, et al (2000) Protein kinase A encoded by *TPK2* regulates dimorphism of *Candida albicans* . Mol Microbiol 35: 386–396. 10652099 10.1046/j.1365-2958.2000.01705.x

[49] Braun BR, Johnson AD (1997) Control of filament formation in *Candida albicans* by the transcriptional repressor *TUP1* . Science 277: 105–109. 9204892 10.1126/science.277.5322.105

[50] Johnston DA, Tapia AL, Eberle KE, Palmer GE (2013) Three prevacuolar compartment Rab GTPases impact *Candida albicans* hyphal growth. Eukaryot Cell 12: 1039–1050. 10.1128/EC.00359-12 23709183 PMC3697461

[51] Piispanen AE, Bonnefoi O, Carden S, Deveau A, Bassilana M, et al (2011) Roles of Ras1 membrane localization during *Candida albicans* hyphal growth and farnesol response. Eukaryot Cell 10: 1473–1484. 10.1128/EC.05153-11 21908593 PMC3209056

[52] Piispanen AE, Grahl N, Hollomon JM, Hogan DA (2013) Regulated proteolysis of *Candida albicans* Ras1 is involved in morphogenesis and quorum sensing regulation. Mol Microbiol 89: 166–178. 10.1111/mmi.12268 23692372 PMC3782256

[53] Visarius TM, Stucki JW, Lauterburg BH (1999) Inhibition and stimulation of long-chain fatty acid oxidation by chloroacetaldehyde and methylene blue in rats. J Pharmacol Exp Ther 289: 820–824. 10215658

[54] Hardie DG (2011) AMP-activated protein kinase: an energy sensor that regulates all aspects of cell function. Genes Dev 25: 1895–1908. 10.1101/gad.17420111 21937710 PMC3185962

[55] Hardie DG, Ross FA, Hawley SA (2012) AMPK: a nutrient and energy sensor that maintains energy homeostasis. Nat Rev Mol Cell Biol 13: 251–262. 10.1038/nrm3311 22436748 PMC5726489

[56] Celenza JL, Eng FJ, Carlson M (1989) Molecular analysis of the *SNF4* gene of *Saccharomyces cerevisiae*: evidence for physical association of the SNF4 protein with the SNF1 protein kinase. Mol Cell Biol 9: 5045–5054. 2481228 10.1128/mcb.9.11.5045-5054.1989 PMC363656

[57] Kanthakumar K, Taylor G, Tsang KW, Cundell DR, Rutman A, et al (1993) Mechanisms of action of *Pseudomonas aeruginosa* pyocyanin on human ciliary beat in vitro. Infect Immun 61: 2848–2853. 8390405 10.1128/iai.61.7.2848-2853.1993 PMC280930

[58] Enloe B, Diamond A, Mitchell AP (2000) A single-transformation gene function test in diploid *Candida albicans* . J Bacteriol 182: 5730–5736. 11004171 10.1128/jb.182.20.5730-5736.2000 PMC94694

[59] Shapiro RS, Uppuluri P, Zaas AK, Collins C, Senn H, et al (2009) Hsp90 orchestrates temperature-dependent *Candida albicans* morphogenesis via Ras1-PKA signaling. Curr Biol 19: 621–629. 10.1016/j.cub.2009.03.017 19327993 PMC2735497

[60] Chautard H, Jacquet M, Schoentgen F, Bureaud N, Benedetti H (2004) Tfs1p, a member of the PEBP family, inhibits the Ira2p but not the Ira1p Ras GTPase-activating protein in *Saccharomyces cerevisiae* . Eukaryot Cell 3: 459–470. 15075275 10.1128/EC.3.2.459-470.2004 PMC387632

[61] Harashima T, Anderson S, Yates JR 3rd, Heitman J (2006) The kelch proteins Gpb1 and Gpb2 inhibit Ras activity via association with the yeast RasGAP neurofibromin homologs Ira1 and Ira2. Mol Cell 22: 819–830. 16793550 10.1016/j.molcel.2006.05.011

[62] Aun A, Tamm T, Sedman J (2013) Dysfunctional mitochondria modulate cAMP-PKA signaling and filamentous and invasive growth of *Saccharomyces cerevisiae* . Genetics 193: 467–481. 10.1534/genetics.112.147389 23172851 PMC3567737

[63] O'Meara TR, Veri AO, Ketela T, Jiang B, Roemer T, et al (2015) Global analysis of fungal morphology exposes mechanisms of host cell escape. Nat Commun 6: 6741 10.1038/ncomms7741 25824284 PMC4382923

[64] Brooks GA, Hittelman KJ, Faulkner JA, Beyer RE (1971) Temperature, skeletal muscle mitochondrial functions, and oxygen debt. Am J Physiol 220: 1053–1059. 4323901 10.1152/ajplegacy.1971.220.4.1053

[65] Dufour S, Rousse N, Canioni P, Diolez P (1996) Top-down control analysis of temperature effect on oxidative phosphorylation. Biochem J 314 (Pt 3): 743–751. 8615765 10.1042/bj3140743 PMC1217120

[66] Ali SS, Marcondes MC, Bajova H, Dugan LL, Conti B (2010) Metabolic depression and increased reactive oxygen species production by isolated mitochondria at moderately lower temperatures. J Biol Chem 285: 32522–32528. 10.1074/jbc.M110.155432 20716522 PMC2952254

[67] Swegert CV, Dave KR, Katyare SS (1999) Effect of aluminium-induced Alzheimer like condition on oxidative energy metabolism in rat liver, brain and heart mitochondria. Mech Ageing Dev 112: 27–42. 10656181 10.1016/s0047-6374(99)00051-2

[68] Morales DK, Jacobs NJ, Rajamani S, Krishnamurthy M, Cubillos-Ruiz JR, et al (2010) Antifungal mechanisms by which a novel *Pseudomonas aeruginosa* phenazine toxin kills *Candida albicans* in biofilms. Mol Microbiol 78: 1379–1392. 10.1111/j.1365-2958.2010.07414.x 21143312 PMC3828654

[69] Dietrich LE, Okegbe C, Price-Whelan A, Sakhtah H, Hunter RC, et al (2013) Bacterial community morphogenesis is intimately linked to the intracellular redox state. J Bacteriol 195: 1371–1380. 10.1128/JB.02273-12 23292774 PMC3624522

[70] Shin SY, Kim TH, Wu H, Choi YH, Kim SG (2014) SIRT1 activation by methylene blue, a repurposed drug, leads to AMPK-mediated inhibition of steatosis and steatohepatitis. Eur J Pharmacol 727: 115–124. 10.1016/j.ejphar.2014.01.035 24486702

[71] Mihaylova MM, Shaw RJ (2011) The AMPK signalling pathway coordinates cell growth, autophagy and metabolism. Nat Cell Biol 13: 1016–1023. 10.1038/ncb2329 21892142 PMC3249400

[72] Vyas VK, Barrasa I, Fink GR (2015) A *Candida albicans* CRISPR system permits genetic engineering of essential genes and gene families. Science Advances 1. 10.1126/sciadv.1500248 PMC4428347 25977940

[73] Zhang F, Pracheil T, Thornton J, Liu Z (2013) Adenosine Triphosphate (ATP) Is a Candidate Signaling Molecule in the Mitochondria-to-Nucleus Retrograde Response Pathway. Genes (Basel) 4: 86–100. 24605246 10.3390/genes4010086 PMC3899953

[74] Bao Y, Ledderose C, Seier T, Graf AF, Brix B, et al (2014) Mitochondria regulate neutrophil activation by generating ATP for autocrine purinergic signaling. J Biol Chem 289: 26794–26803. 10.1074/jbc.M114.572495 25104353 PMC4175322

[75] Burnstock G (2006) Historical review: ATP as a neurotransmitter. Trends Pharmacol Sci 27: 166–176. 16487603 10.1016/j.tips.2006.01.005

[76] Colombo S, Paiardi C, Pardons K, Winderickx J, Martegani E (2014) Evidence for adenylate cyclase as a scaffold protein for Ras2-Ira interaction in *Saccharomyces cerevisie* . Cell Signal 26: 1147–1154. 10.1016/j.cellsig.2014.02.001 24518043

[77] Hlavata L, Nystrom T (2003) Ras proteins control mitochondrial biogenesis and function in Saccharomyces cerevisiae. Folia Microbiol (Praha) 48: 725–730. 15058183 10.1007/BF02931505

[78] Belotti F, Tisi R, Paiardi C, Rigamonti M, Groppi S, et al (2012) Localization of Ras signaling complex in budding yeast. Biochim Biophys Acta 1823: 1208–1216. 10.1016/j.bbamcr.2012.04.016 22575457

[79] Galello F, Moreno S, Rossi S (2014) Interacting proteins of protein kinase A regulatory subunit in *Saccharomyces cerevisiae* . J Proteomics 109: 261–275. 10.1016/j.jprot.2014.07.008 25065647

[80] Hall RA, De Sordi L, Maccallum DM, Topal H, Eaton R, et al (2010) CO(2) acts as a signalling molecule in populations of the fungal pathogen *Candida albicans* . PLoS Pathog 6: e1001193 10.1371/journal.ppat.1001193 21124988 PMC2987819

[81] Xu XL, Lee RT, Fang HM, Wang YM, Li R, et al (2008) Bacterial peptidoglycan triggers *Candida albicans* hyphal growth by directly activating the adenylyl cyclase Cyr1p. Cell Host Microbe 4: 28–39. 10.1016/j.chom.2008.05.014 18621008

[82] Wilson RB, Davis D, Mitchell AP (1999) Rapid hypothesis testing with *Candida albicans* through gene disruption with short homology regions. J Bacteriol 181: 1868–1874. 10074081 10.1128/jb.181.6.1868-1874.1999 PMC93587

[83] Murad AM, Lee PR, Broadbent ID, Barelle CJ, Brown AJ (2000) CIp10, an efficient and convenient integrating vector for *Candida albicans* . Yeast 16: 325–327. 10669870 10.1002/1097-0061(20000315)16:4<325::AID-YEA538>3.0.CO;2-#

[84] Miller L (2010) Analyzing gels and western blots with ImageJ.

[85] Geiss GK, Bumgarner RE, Birditt B, Dahl T, Dowidar N, et al (2008) Direct multiplexed measurement of gene expression with color-coded probe pairs. Nat Biotechnol 26: 317–325. 10.1038/nbt1385 18278033

[86] Warnes GR, Bolker B, Bonebakker L, Gentleman R, Huber W, et al (2014) R: A language and environment for statistical computing 2.15.0 ed. Vienna, Austria: R Foundation for Statistical Computing.

[87] Kestler HA, Muller A, Kraus JM, Buchholz M, Gress TM, et al (2008) VennMaster: area-proportional Euler diagrams for functional GO analysis of microarrays. BMC Bioinformatics 9: 67 10.1186/1471-2105-9-67 18230172 PMC2335321

[88] Micallef L, Rodgers P (2014) eulerAPE: drawing area-proportional 3-Venn diagrams using ellipses. PLoS One 9: e101717 10.1371/journal.pone.0101717 25032825 PMC4102485

