# Supplementary figures and images for: Mitochondrial Activity and Cyr1 Are Key Regulators of Ras1 Activation of C. albicans Virulence Pathways
Source: PLoS Pathog. 2015 Aug 28;11(8):e1005133. doi: 10.1371/journal.ppat.1005133 (PMC4552728; doi:10.1371/journal.ppat.1005133)

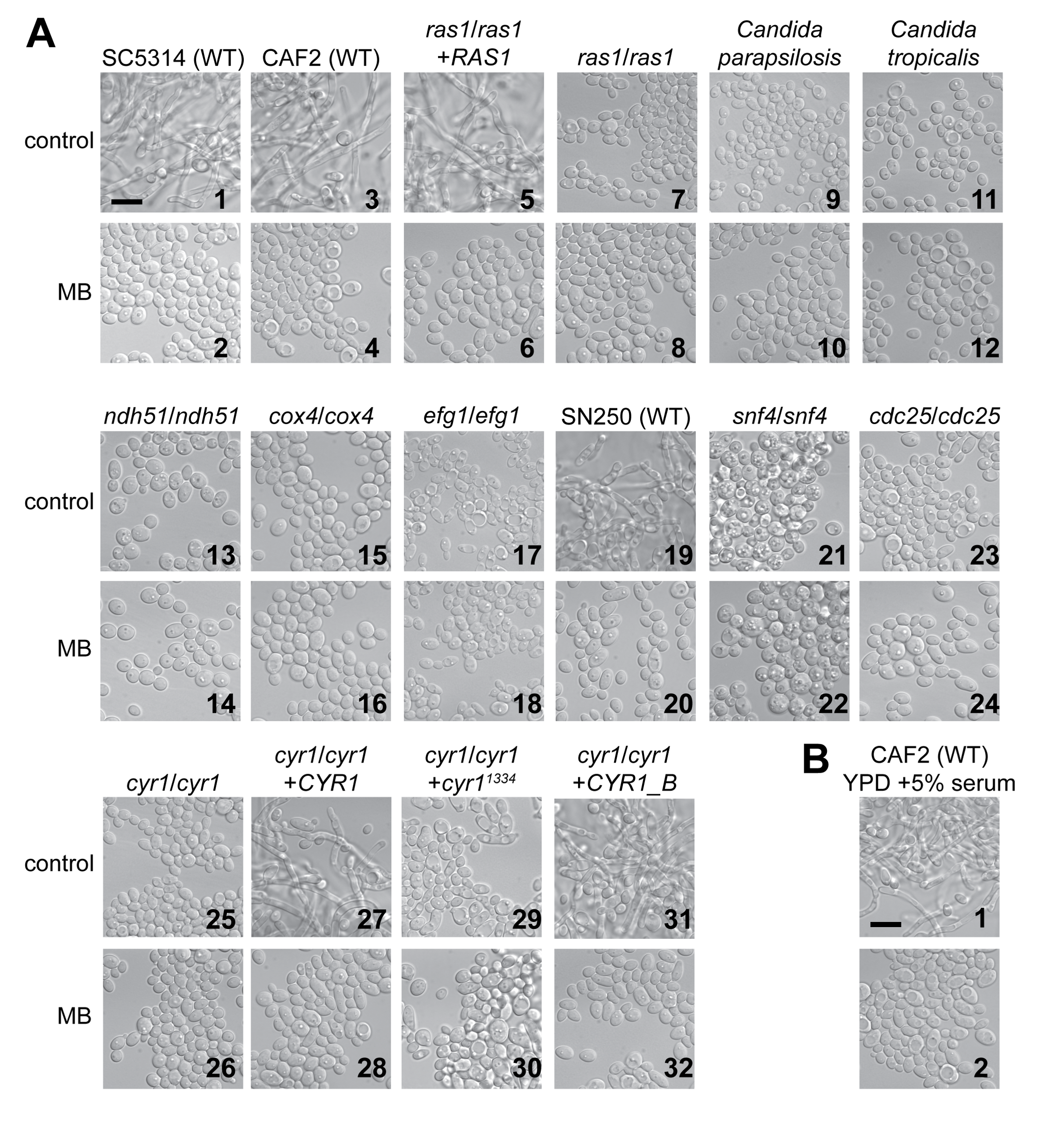

Supplement: S1 Fig — Cells were grown for 24 h on (A) YNBAGNP or (B) YPD +5% serum at 37°C in spot colonies before imaging. Microscopy pictures of CAF2 in (A) are identical to images shown in Fig 4C. Scale bar = 10 μm. (TIF) [file ppat.1005133.s001.tif]

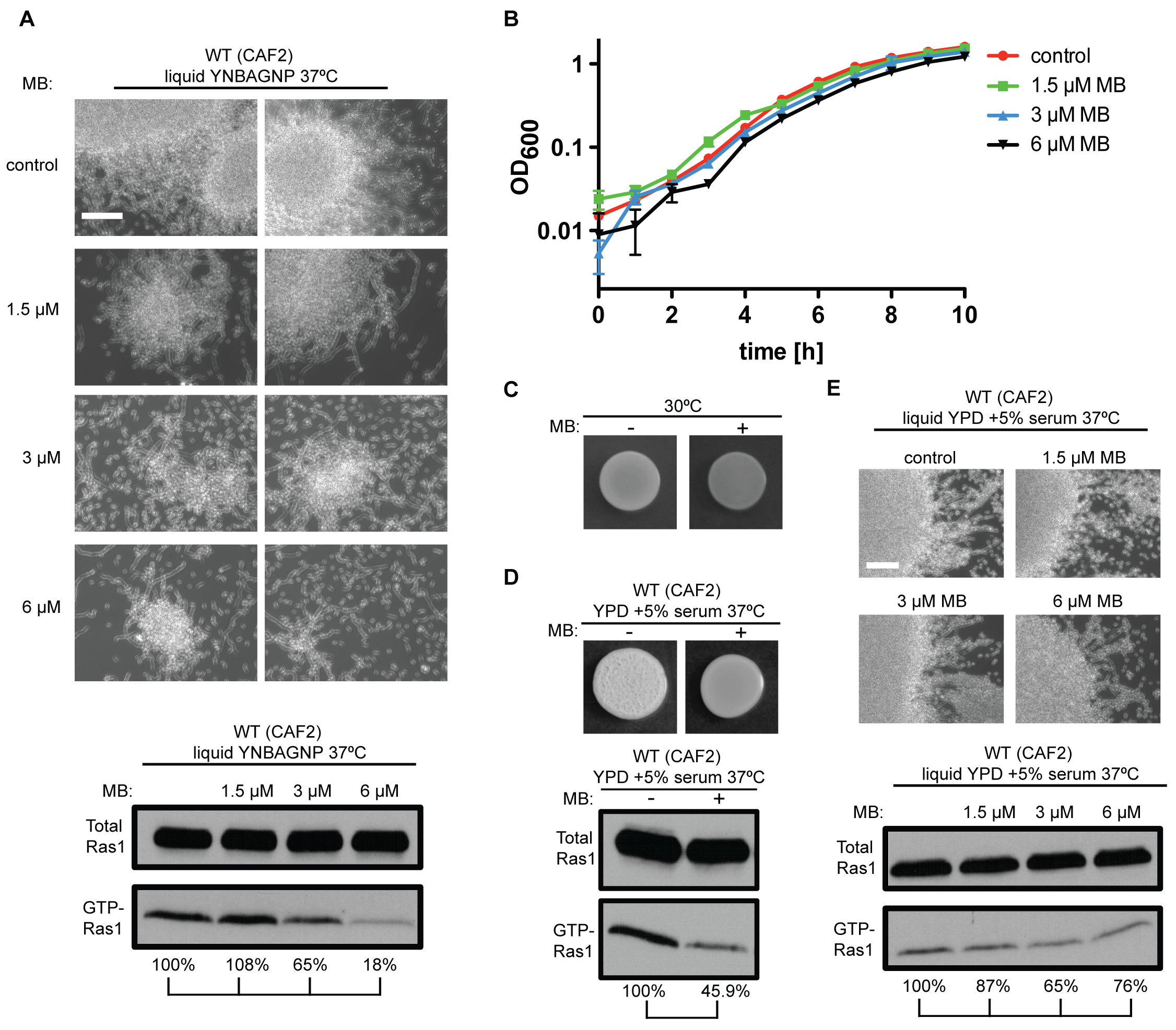

Supplement: S2 Fig — (A) Microscopy images and Western blot analysis of WT (CAF2) grown in liquid YNBAGNP media for 12 hours at 37°C. Scale bar = 100 μm. (B) Growth of C. albicans strain NRG1-OE in the presence of different concentrations of MB. Data represents the mean and SD of three biological replicates grown in liquid YNBAGNP at 37°C. (C) Smooth colony morphology (consisting of yeast only) in the presence and absence of MB at yeast growth conditions (30°C; YNBGP; 24 h). (D) WT (CAF2) grown on YPD +5% serum with and without MB (see S1B Fig for cellular morphology). Western blot analysis of total Ras1 and GTP-Ras1 levels shown. Cells were grown for 24 h at 37°C (E) Microscopy images and Western blot analysis of WT (CAF2) grown in liquid YPD +5% serum media for 12 hours at 37°C. Scale bar = 100 μm. (A), (D), and (E) Percent of the GTP-Ras1/total Ras1 ratio compared to WT control conditions is shown. (TIF) [file ppat.1005133.s002.tif]

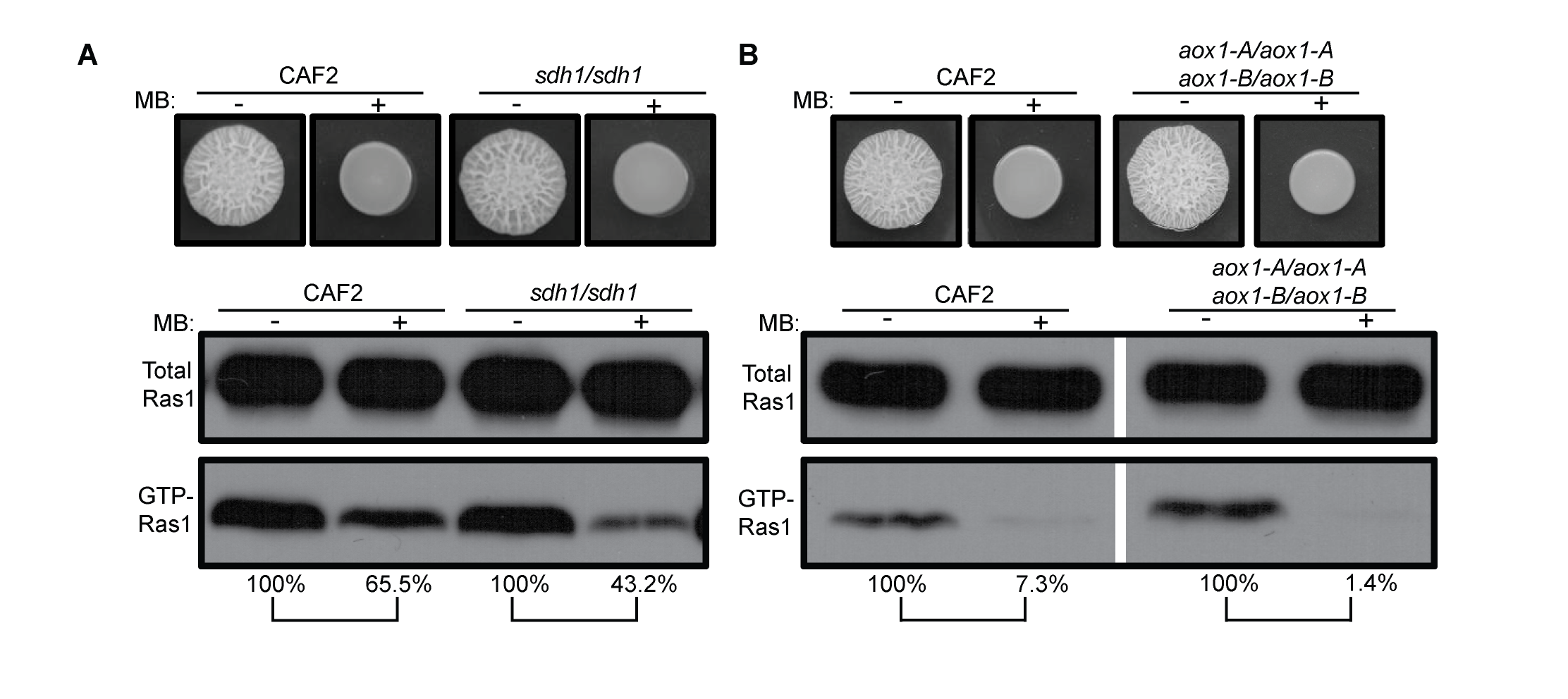

Supplement: S3 Fig — Colony morphology and western blot analysis of the WT (CAF2), sdh1/sdh1 (complex II), and aox1/aox1 aox2/aox2 (alternative oxidase) strains grown on YNBAGNP for 24 h at 37°C with and without MB are shown. (B) Western samples were run on the same gel. Percent of the GTP-Ras1/total Ras1 ratio compared to WT control conditions is shown. (TIF) [file ppat.1005133.s003.tif]

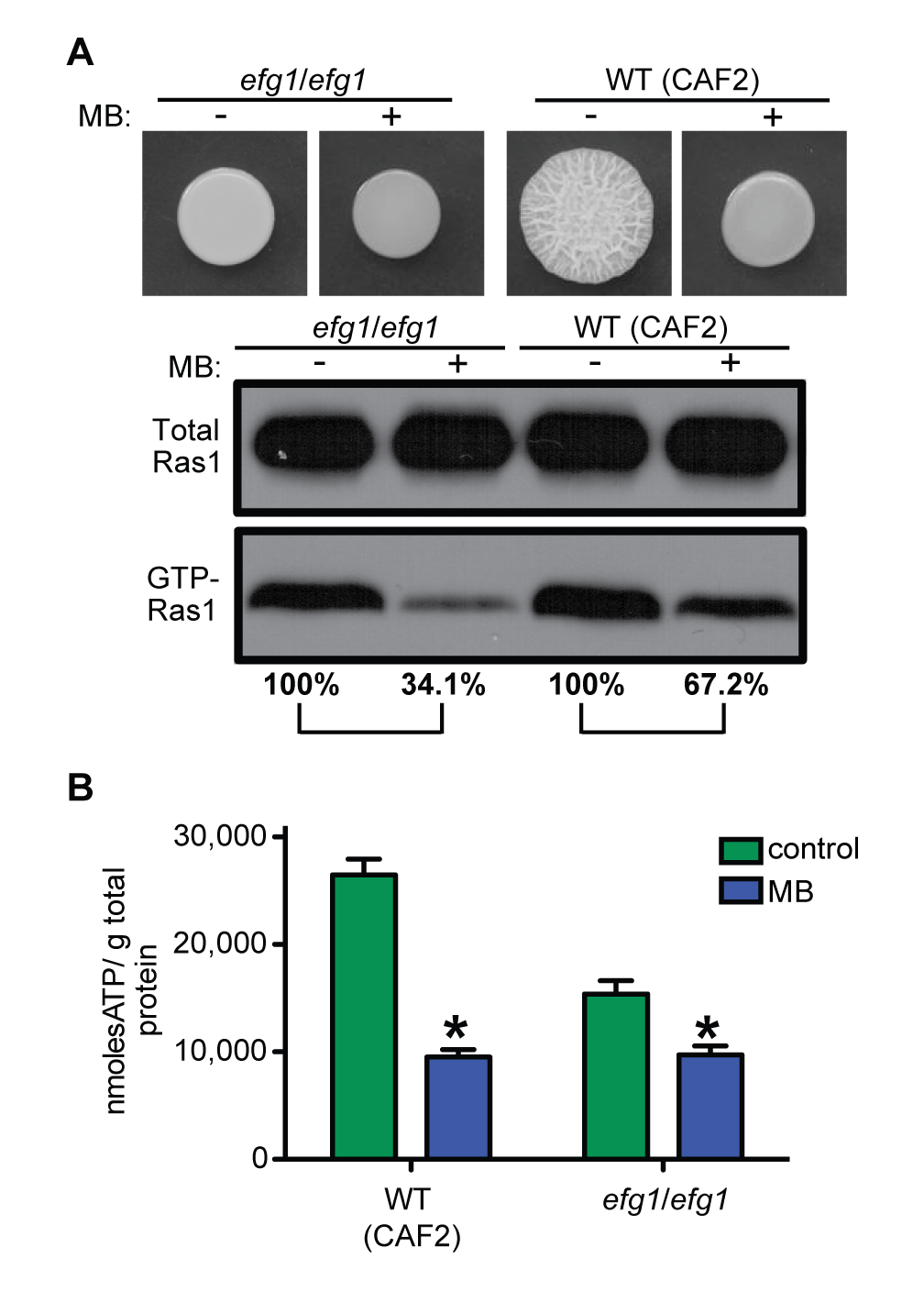

Supplement: S4 Fig — (A) Western blot analysis and (B) intracellular ATP measurements comparing WT (CAF2) with the yeast locked efg1/efg1 strain are shown. Cells were grown on YNBAGNP for 24 h at 37°C with and without MB. (A) Percent of the GTP-Ras1/total Ras1 ratio compared to control conditions is reported. (B) Mean ± SD are shown. *p<0.05. (TIF) [file ppat.1005133.s004.tif]

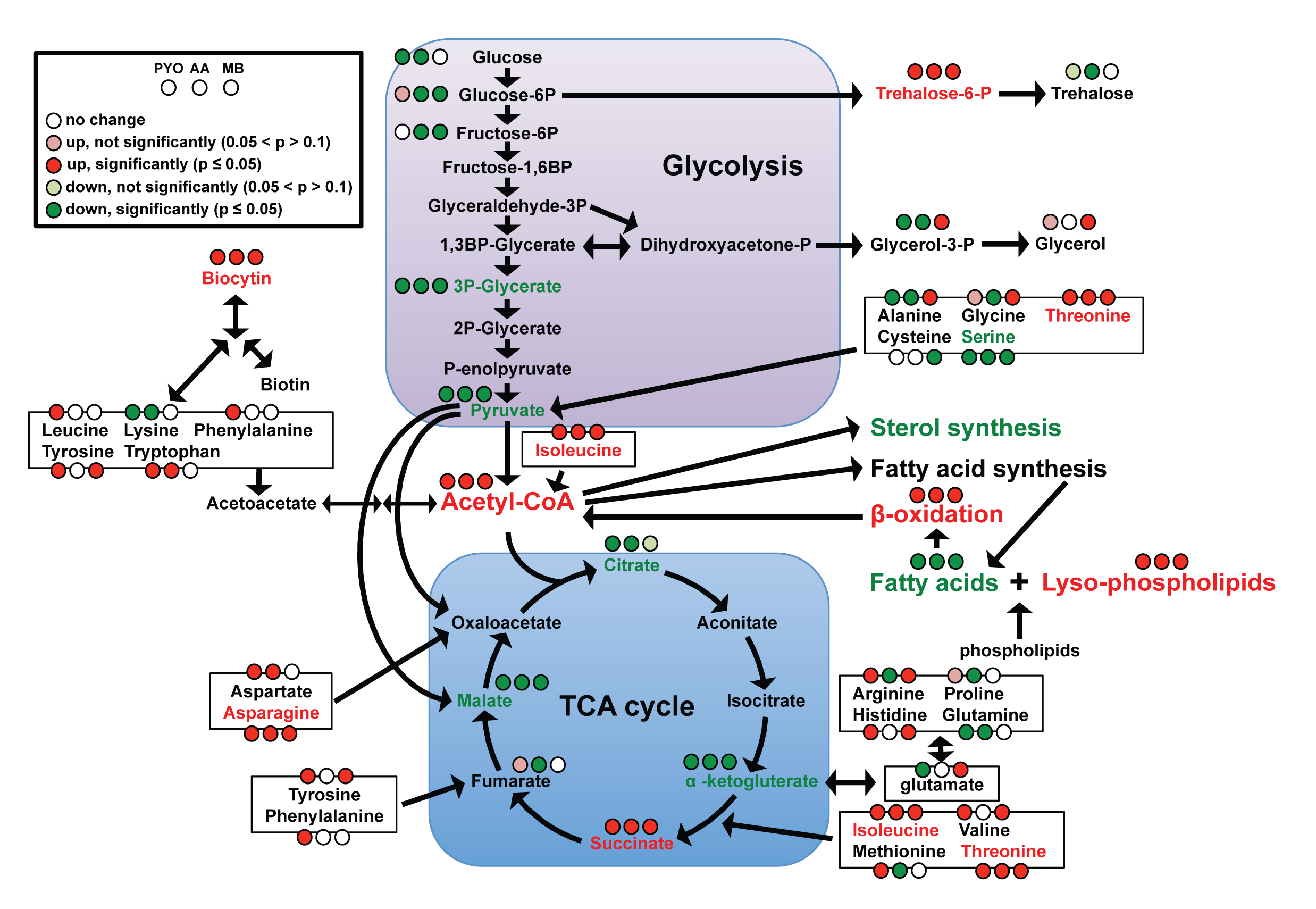

Supplement: S5 Fig — Data represent a subset of metabolites changed. See S1 Table for the complete data set. (TIF) [file ppat.1005133.s005.tif]

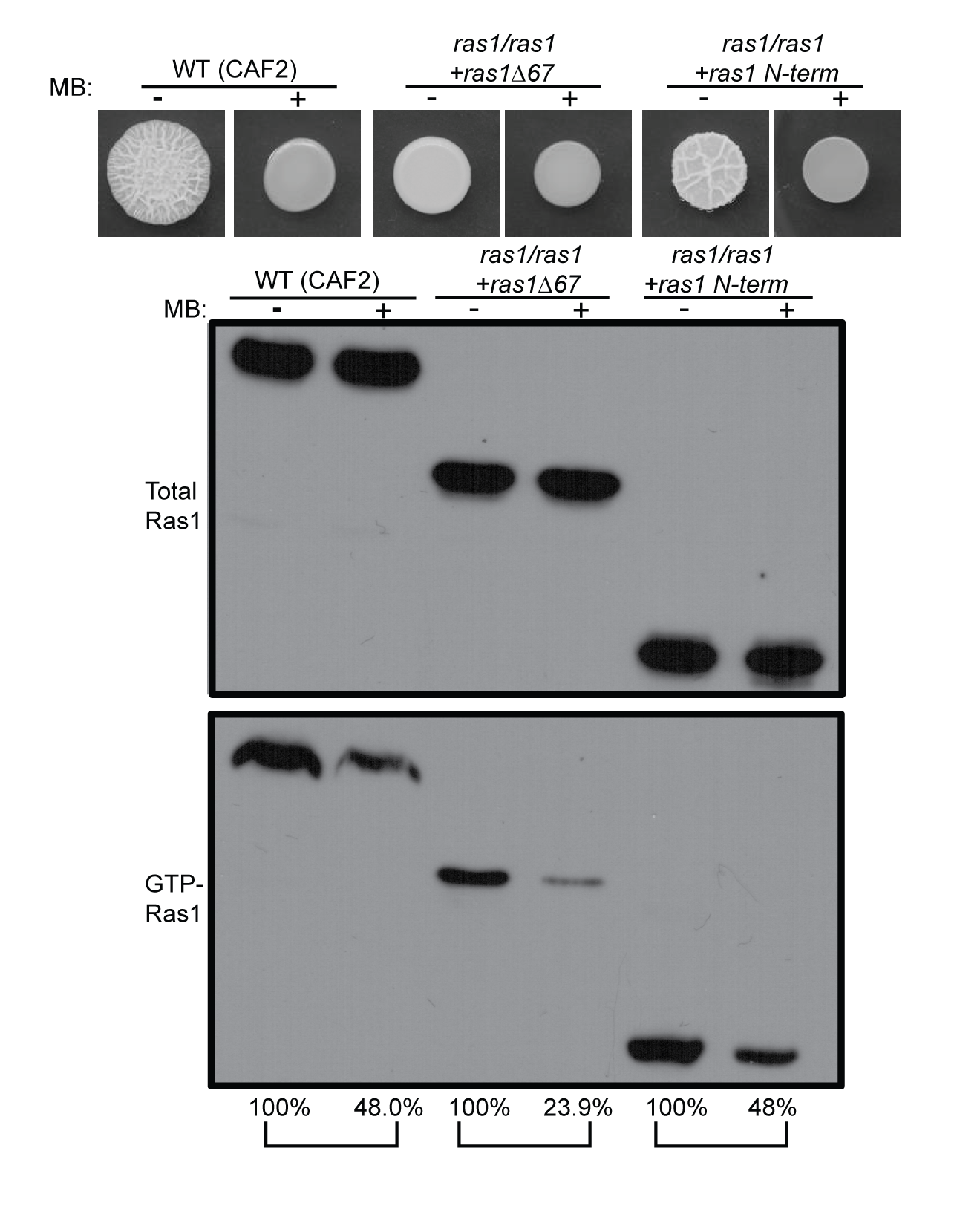

Supplement: S6 Fig — Colony morphology and western blot analysis of WT (CAF2), ras1/ras1 +ras1Δ67, and ras1/ras1 +ras1N-term are shown. Percent of the GTP-Ras1/total Ras1 ratio compared to WT control conditions is shown. Cells were grown on YNBAGNP for 24 h at 37°C with and without MB. (TIF) [file ppat.1005133.s006.tif]

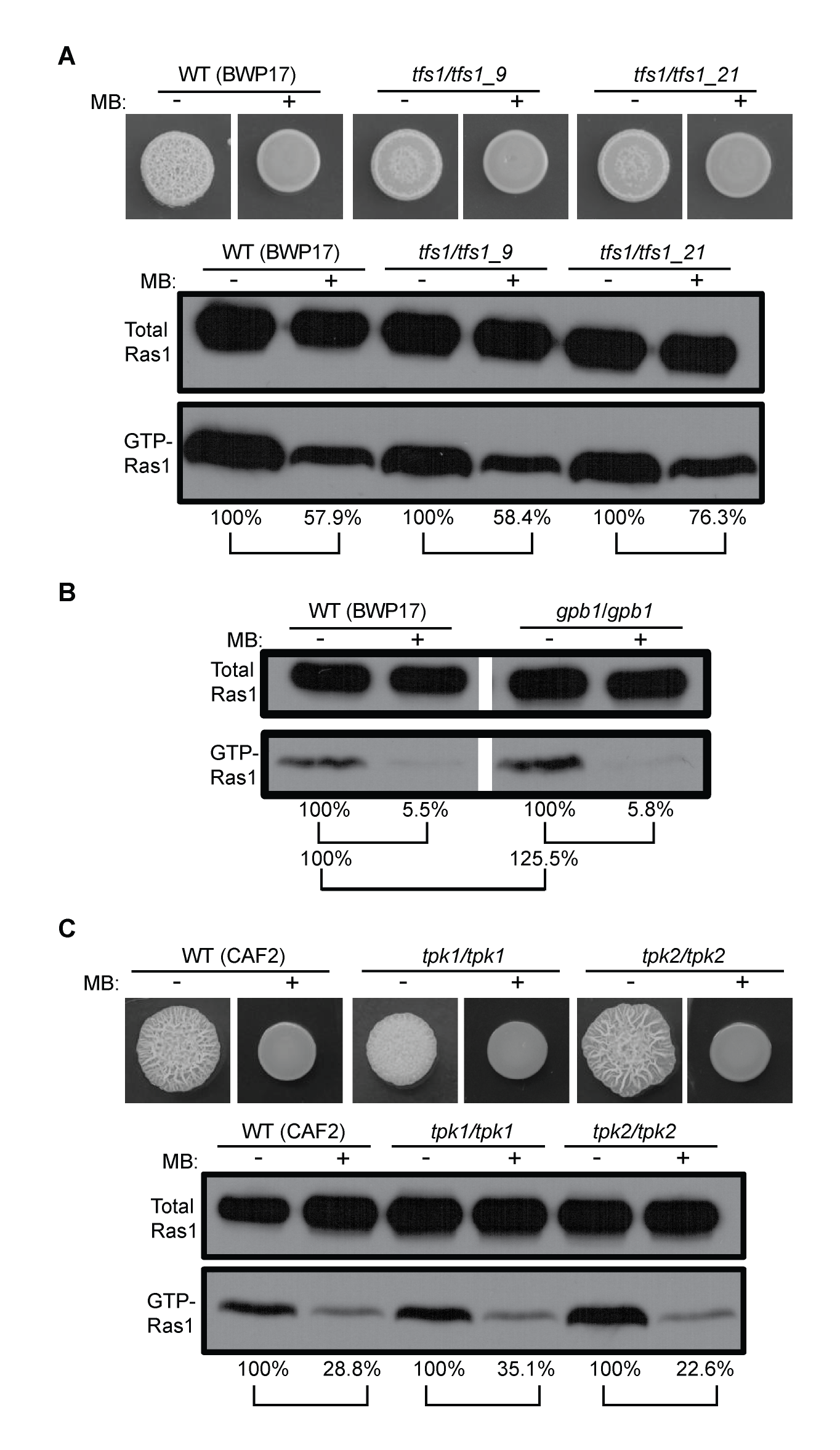

Supplement: S7 Fig — (A) (B) (C) Colony morphology and western blot analysis of total Ras1 and GTP-Ras1 in the WT (BWP17, CAF2), tfs1/tfs1_9, tfs1/tfs1_21, gpb1/gpb1, tpk1/tpk1, and tpk2/tpk2 strains are shown. Cells were grown on YNBAGNP for 24 h at 37°C with and without MB. (B) Western samples were run on the same gel. Percent of the GTP-Ras1/total Ras1 ratio compared to control conditions is reported. Percent of the GTP-Ras1/total Ras1 ratio compared to WT control conditions is shown. (TIF) [file ppat.1005133.s007.tif]
