# Supplementary material for: Mitochondrial Activity and Cyr1 Are Key Regulators of Ras1 Activation of C. albicans Virulence Pathways
Source: PLoS Pathog. 2015 Aug 28;11(8):e1005133. doi: 10.1371/journal.ppat.1005133 (PMC4552728; doi:10.1371/journal.ppat.1005133)
Supplement: S2 Table — (DOCX) [file ppat.1005133.s009.docx]

**S2 Table. Strains used in this study.**

| **Strain name** | **genotype** | **lab stock #** | **source** |
| --- | --- | --- | --- |
| SC5314 | prototrophic clinical isolate | DH35 | [1] |
| CAF2 | *URA3/ura3::λimm434 IRO1/ira1::λimm434* | DH331 | [2] |
| BWP17 | *ura3::λimm434/ura3::λimm434 arg4::hisG/arg4::hisG his1::hisG/his1::hisG* | DH1505 | [3] |
| BWP17 prototroph | *ura3::λimm434/URA3 arg4::hisG/ARG4 his1::hisG/HIS1* | DH1671  (CEC17) | from  Martine Raymond |
| *ras1*/*ras1* ura- | *ura3::λimm434/ura3::λimm434 ras1::hisG/ras1::hisG* | DH482 | [4] |
| *ras1*/*ras1* | *ura3::λimm434/ura3::λimm434 ras1::hisG/ras1::hisG:URA3* | DH483 | [4] |
| *ras1*/*ras1* +*RAS1* | *ura3::λimm434/ura3::λimm434 ras1::hisG/ras1::hisG:RAS1-URA3* | DH1385 | [5] |
| *ras1*Δ67 | *ura3::λimm434/ura3::λimm434 ras1::hisG/ras1::hisG:ras1Δ67-URA3* | DH1941 | [6] |
| *ras1*/*ras1* +*ras1 N-term* | *ura3::λimm434/ura3::λimm434 ras1::hisG/ras1::hisG:ras1 N-term-URA3* | DH1761 | This study |
| *cyr1*/*cyr1* | *ura3::λimm434/ura3::λimm434::URA3 arg4::hisG/arg4::hisG his1::hisG/his1::hisG cyr1::HIS1/cyr1::ARG4* | DH2185 | This study |
| *cyr1*/*cyr1* +*CYR1* | *ura3::λimm434/ura3::λimm434::URA3-CYR1 arg4::hisG/arg4::hisG his1::hisG/his1::hisG cyr1::HIS1/cyr1::ARG4* | DH2186 | This study |
| *cyr1*/*cyr1* +*CYR1*_B | *ura3::λ imm434/ura3::λimm434::URA3-CYR1 cyr1::hisG/cyr1::hisG* | DH1690  (RH21) | [7] |
| *cyr1*/*cyr1* +*cyr1*^1334^ | *ura3::λ imm434/ura3::λimm434::URA3-cyr1-1334 cyr1::hisG/cyr1::hisG* | DH1692  (RH26) | [7] |
| *tpk1*/*tpk1* | *ura3::λimm434/ura3::λimm434 arg4::hisG/arg4::hisG his1::hisG/his1::hisG tpk1::Tn7-UAU1/tpk1Tn7-ARG4* | DH1500 | [8] |
| *tpk2*/*tpk2* | *ura3::λimm434/ura3::λimm434 arg4::hisG/arg4::hisG his1::hisG/his1::hisG tpk2::Tn7-UAU1/tpk2Tn7-ARG4* | DH1498 | [8] |
| *cdc25*/*cdc25* | *ura3::λimm434/ura3::λimm434 arg4::hisG/arg4::hisG his1::hisG/his1::hisG cdc25::HIS1/cdc25::ARG4* | DH2064 | This study |
| *ira2*/*ira2* | *ura3::λimm434/ura3::λimm434::URA3 arg4::hisG/arg4::hisG his1::hisG/his1::hisG ira2::HIS1/ira2::ARG4* | DH2240 | This study |
| *tfs1*/*tfs1*_9 | *ura3::λimm434/ura3::λimm434 arg4::hisG/arg4::hisG his1::hisG/his1::hisG tfs1::HIS1/tfs1::ARG4* | DH2427 | This study |
| *tfs1*/*tfs1*_21 | *ura3::λimm434/ura3::λimm434 arg4::hisG/arg4::hisG his1::hisG/his1::hisG tfs1::HIS1/tfs1::ARG4* | DH2428 | This study |
| *gpb1*/*gpb1* | *ura3::λimm434/ura3::λimm434 arg4::hisG/arg4::hisG his1::hisG/his1::hisG gpb1::HIS1/gpb1::ARG4* | DH2446 | This study |
| *ndh51*/*ndh51* | *ura3Δ::λimm434/ura3Δ::λimm434 his1::hisG/his1::hisG arg4::hisG/arg4::hisG ndh51::ARG4/ndh51::URA3* | DH1618  (JM02) | [9] |
| *sdh1*/*sdh1* | *ura3::λimm434/ura3::λimm434 arg4::hisG/arg4::hisG his1::hisG/his1::hisG sdh1::URA3/sdh1::ARG4* | DH1871 | This study |
| *aox1-A*/*aox1-A*  *aox1-B*/*aox1-B* | *ura3::λimm434/ura3::λimm434 aox1-b-aox1-a::hisG/aox1-b-aox1-a::hisG* | DH1562  (WH323) | [10] |
| SN250 | *ura3::λimm434::URA3/ura3::λimm434 iro1::IRO1/iro1::λimm434 his1::hisG/his1::hisG leu2/leu2 arg4/arg4* | DH2424 | [11] |
| *cox4*/*cox4* | *ura3::λimm434::URA3/ura3::λimm434 iro1::IRO1/iro1::λimm434 his1::hisG/his1::hisG leu2/leu2 arg4/arg4 cox4::HIS1/cox4::LEU2* | DH2425 | [11] |
| *snf4*/*snf4* | *ura3::λimm434::URA3/ura3::λimm434 iro1::IRO1/iro1::λimm434 his1::hisG/his1::hisG leu2/leu2 arg4/arg4 snf4::HIS1/snf4::LEU2* | DH2426 | [11] |
| TT21 | *ade2::hisG/ade2::hisG ura3::λimm434/ura3::λimm434::URA3-tetO-ENO1/eno1::ENO1-tetR-ScHAP4AD-3XHA-ADE2* | DH2314 | [12] |
| *NRG1-OE* | *ade2::hisG/ade2::hisG ura3::λimm434/ura3::λimm434::URA3-tet-O-NRG1 ENO1/eno1::ENO1-tetR-ScHAP4AD-3XHA-ADE2* | DH2315 | [13] |
| *UME6-OE* | *ade2::hisG/ade2::hisG ura3::λimm434/ura3::λimm434::URA3-tet-O-UME6 ENO1/eno1::ENO1-tetR-ScHAP4AD-3XHA-ADE2* | DH2317 | [12] |
| *efg1*/*efg1* | *ura3*:: *λimm434*/*ura3*:: *λimm434 efg1*::*hisG*/*efg1*::*hisG-URA3-hisG* | DH116  (HLC52) | [14] |
| *tup1/tup1* | *tup1::hisG/tup1::p405-URA3 ura3/ura3* | DH36 | [15] |
| *ssn3*/*ssn3* | *ura3::λimm434/ura3::λimm434 arg4::hisG/arg4::hisG his1::hisG/his1::hisG::pHIS1 ssn3::Tn7-UAU1/ssn3Tn7-URA3* | DH1969 | [16] |
| *ssn3*/*ssn3* +*SSN3* | *ura3::λimm434/ura3::λimm434 arg4::hisG/arg4::hisG his1::hisG/his1::hisG::pHIS1-SSN3 ssn3::Tn7-UAU1/ssn3Tn7-URA3* | DH2073 | [16] |
| *Candida parapsilosis* | prototrophic clinical isolate | DH1989 | [17] |
| *Candida tropicalis* | prototrophic clinical isolate | DH1988 | [17] |

**References**

1. Gillum AM, Tsay EY, Kirsch DR (1984) Isolation of the *Candida albicans* gene for orotidine-5'-phosphate decarboxylase by complementation of *S. cerevisiae ura3* and *E. coli pyrF* mutations. Mol Gen Genet 198: 179-182.

2. Fonzi WA, Irwin MY (1993) Isogenic strain construction and gene mapping in *Candida albicans*. Genetics 134: 717-728.

3. Wilson RB, Davis D, Mitchell AP (1999) Rapid hypothesis testing with *Candida albicans* through gene disruption with short homology regions. J Bacteriol 181: 1868-1874.

4. Leberer E, Harcus D, Dignard D, Johnson L, Ushinsky S, et al. (2001) Ras links cellular morphogenesis to virulence by regulation of the MAP kinase and cAMP signalling pathways in the pathogenic fungus *Candida albicans*. Mol Microbiol 42: 673-687.

5. Piispanen AE, Bonnefoi O, Carden S, Deveau A, Bassilana M, et al. (2011) Roles of Ras1 membrane localization during *Candida albicans* hyphal growth and farnesol response. Eukaryot Cell 10: 1473-1484.

6. Piispanen AE, Grahl N, Hollomon JM, Hogan DA (2013) Regulated proteolysis of *Candida albicans* Ras1 is involved in morphogenesis and quorum sensing regulation. Mol Microbiol 89: 166-178.

7. Hall RA, De Sordi L, Maccallum DM, Topal H, Eaton R, et al. (2010) CO(2) acts as a signalling molecule in populations of the fungal pathogen *Candida albicans*. PLoS Pathog 6: e1001193.

8. Blankenship JR, Fanning S, Hamaker JJ, Mitchell AP (2010) An extensive circuitry for cell wall regulation in *Candida albicans*. PLoS Pathog 6: e1000752.

9. McDonough JA, Bhattacherjee V, Sadlon T, Hostetter MK (2002) Involvement of *Candida albicans* NADH dehydrogenase complex I in filamentation. Fungal Genet Biol 36: 117-127.

10. Huh WK, Kang SO (2001) Characterization of the gene family encoding alternative oxidase from *Candida albicans*. Biochem J 356: 595-604.

11. Noble SM, French S, Kohn LA, Chen V, Johnson AD (2010) Systematic screens of a *Candida albicans* homozygous deletion library decouple morphogenetic switching and pathogenicity. Nat Genet 42: 590-598.

12. Johnston DA, Tapia AL, Eberle KE, Palmer GE (2013) Three prevacuolar compartment Rab GTPases impact *Candida albicans* hyphal growth. Eukaryot Cell 12: 1039-1050.

13. Peters BM, Palmer GE, Nash AK, Lilly EA, Fidel PL, Jr., et al. (2014) Fungal morphogenetic pathways are required for the hallmark inflammatory response during *Candida albicans* vaginitis. Infect Immun 82: 532-543.

14. Lo HJ, Kohler JR, DiDomenico B, Loebenberg D, Cacciapuoti A, et al. (1997) Nonfilamentous *C. albicans* mutants are avirulent. Cell 90: 939-949.

15. Braun BR, Johnson AD (1997) Control of filament formation in *Candida albicans* by the transcriptional repressor *TUP1*. Science 277: 105-109.

16. Lindsay AK, Morales DK, Liu Z, Grahl N, Zhang A, et al. (2014) Analysis of *Candida albicans* mutants defective in the Cdk8 module of mediator reveal links between metabolism and biofilm formation. PLoS Genet 10: e1004567.

17. Alex D, Gay-Andrieu F, May J, Thampi L, Dou D, et al. (2012) Amino acid-derived 1,2-benzisothiazolinone derivatives as novel small-molecule antifungal inhibitors: identification of potential genetic targets. Antimicrob Agents Chemother 56: 4630-4639.
