# Supplementary material for: Mitochondrial Activity and Cyr1 Are Key Regulators of Ras1 Activation of C. albicans Virulence Pathways
Source: PLoS Pathog. 2015 Aug 28;11(8):e1005133. doi: 10.1371/journal.ppat.1005133 (PMC4552728; doi:10.1371/journal.ppat.1005133)
Supplement: S3 Table — (DOCX) [file ppat.1005133.s010.docx]

**S3 Table. Plasmids used in this study.**

| **plasmid** | **description** | **lab stock #** | **source** |
| --- | --- | --- | --- |
| pSM2 | *URA3* integrating plasmid at the *URA3* locus | DH1698 | [1] |
| pSMTC | p*TEF*2, full-length *CYR1* in pSM2 | DH1697 | [1] |
| pGEM-HIS1 | For amplification of *HIS1* marker for knockout | DH1564 | [2] |
| pRS-ARG4ΔSpe1 | For amplification of *ARG4* marker for knockout | DH1567 | [2] |
| pClp10 | *URA3* integrating plasmid at the RP10 locus | DH2423 | [3] |
| pAP13 | p*RAS1*-mcs | DH1717 | [4] |
| pAP13+ras1 N-term | pAP13 with ras1-1-162aa | DH2431 | This study |

**References**

1. Hall RA, De Sordi L, Maccallum DM, Topal H, Eaton R, et al. (2010) CO(2) acts as a signalling molecule in populations of the fungal pathogen *Candida albicans*. PLoS Pathog 6: e1001193.

2. Wilson RB, Davis D, Mitchell AP (1999) Rapid hypothesis testing with *Candida albicans* through gene disruption with short homology regions. J Bacteriol 181: 1868-1874.

3. Murad AM, Lee PR, Broadbent ID, Barelle CJ, Brown AJ (2000) CIp10, an efficient and convenient integrating vector for *Candida albicans*. Yeast 16: 325-327.

4. Deveau A, Piispanen AE, Jackson AA, Hogan DA (2010) Farnesol induces hydrogen peroxide resistance in *Candida albicans* yeast by inhibiting the Ras-cyclic AMP signaling pathway. Eukaryot Cell 9: 569-577.
