# Supplementary material for: Mitochondrial Activity and Cyr1 Are Key Regulators of Ras1 Activation of C. albicans Virulence Pathways
Source: PLoS Pathog. 2015 Aug 28;11(8):e1005133. doi: 10.1371/journal.ppat.1005133 (PMC4552728; doi:10.1371/journal.ppat.1005133)
Supplement: S4 Table — (DOCX) [file ppat.1005133.s011.docx]

**S4 Table. Sequences of primers used in this study.**

| **Primer name** | **sequence** |
| --- | --- |
| ira2_KO_fw | CACATAGACATTATATTTGCTTCATTGTTATTGATAAGAGTTGTTAATTCC**TGTGGAATTGTGAGCGGATA** |
| ira2_KO_rev | CCAAAAGCCTCTAACCTAAAATGAACGTTGTTCTATAATACAAAATTAACTAGC**TTTCCCAGTCACGACGTT** |
| cdc25_KO_fw | GAGCTTGTGTTGATACGCATTGTACAAATATAAGGATAGCTTAGGATACAG**TGTGGAATTGTGAGCGGATA** |
| cdc25_KO_rev | GTGTATTAGAAGTCTGTAGTTCTTCAACAGAAAAGGAAGGATAAGAGTG**TTTCCCAGTCACGACGTT** |
| cyr1_KO_fw | CTCACTCACTCACTCACGTTCAACCTTCAACCCTCAATCTATTTTTACCA**TGTGGAATTGTGAGCGGATA** |
| cyr1_KO_rev | TAGTAGTAATTTTGTTTAAGAATATTTACATGGACCAACCAATGAACCTC**TTTCCCAGTCACGACGTT** |
| SDH1_F2 | CTCCTATTCAGTGATTTTG**TGTGGAATTGTGAGCGGATA** |
| SDH1_R2 | CGTGTACGACATTTATACTG**TTTCCCAGTCACGACGTT** |
| tfs1_KO_fw | CCCCTTTCTCTGCCAATTATATCCCCGAGCAATTATAACATTTTAGGTCC**TGTGGAATTGTGAGCGGATA** |
| tfs1_KO_rev | GACTGATTTCTGGCTCGCCATCTTCATTCTTGGTGTGTGGCAATTTCAAG**TTTCCCAGTCACGACGTT** |
| gpb1_KO_fw | TTGAGATTATCTTTAAACAATATACACCACACGAGCGTTGCAAGAGAGAG**TGTGGAATTGTGAGCGGATA** |
| gpb1_KO_rev | CCCCATAGGAGCTCTTCTATAGGAAACTGTTTCCTCAATTCATCCATATC**TTTCCCAGTCACGACGTT** |
| RAS1delta129BamHI-R | ACTGGATCCTCATCTTACTAATCCATAAAATG |

**bold** = plasmid homologous sequences to amplify selection marker
